# Moisturizer formulations for diabetic foot xerosis: a systematic review and meta-analysis of randomized controlled trials

**DOI:** 10.3389/fcdhc.2026.1922860

**Published:** 2026-09-03

**Authors:** Karin Borgström, Sebastian Björklund, Petri Gudmundsson, Anna Ericsson, Stefan Acosta

**Affiliations:** 1 Department of Biomedical Science, Malmö University, Malmö, Sweden; 2 Biofilms Research Centre for Biointerfaces, Malmö University, Malmö, Sweden; 3 Department of Care Science, Malmö University, Malmö, Sweden; 4 Department of Clinical Sciences, Malmö, Lund University, Malmö, Sweden

**Keywords:** diabetes mellitus, diabetic foot, meta-analysis, skin cream, systematic review, urea

## Abstract

**Background:**

Foot xerosis in people with diabetes increases the risk of skin fissuring, ulceration and complications like amputation, sepsis and death. Preventive skin care is therefore important, yet evidence-based guidance on the most effective formulations remains limited. This systematic review aimed to identify effective ingredients and formulations to treat diabetic foot xerosis.

**Methods:**

A literature search was conducted in Medline, Cinahl, Embase, CENTRAL and Scopus. Randomized controlled trials (RCTs) involving adults with diabetes and foot xerosis (without ulcers) that compared topical formulations were eligible. Reviewers screened studies, extracted data, assessed risk of bias and certainty of evidence in accordance with Cochrane Intervention Review standards. Outcomes included xerosis severity, skin hydration, skin barrier integrity and adverse events. Meta-analyses of RCTs were conducted. The review was registered in PROSPERO (CRD42024579919).

**Results:**

Eleven articles reporting twelve RCTs were included. Functional creams (i.e., base formulations with added ingredients, most commonly humectants, intended to enhance their effect) significantly reduced xerosis severity compared with standard base formulations after both two weeks (standardized mean difference [SMD] -0.58, 95% CI -1.05 to -0.11) and four weeks (SMD -0.30, 95% CI -0.46 to -0.14). The certainty of evidence was moderate. Functional creams significantly increased skin hydration (SMD 0.31, 95% CI 0.11 to 0.52), although the certainty of evidence was low. Creams containing urea were more effective than standard base creams after four weeks (SMD -0.36, 95% CI -0.55 to - 0.17), with high-certainty evidence. Adverse events were rare and mild.

**Conclusions:**

Functional creams, particularly those containing humectants and primarily urea, show greater efficacy than standard bases. However, high-quality randomized controlled trials with long-term outcomes such as risk of foot ulcers are warranted.

**Systematic review registration:**

https://www.crd.york.ac.uk/PROSPERO/view/CRD42024579919, identifier CRD42024579919.

## Introduction

Diabetes mellitus has become one of the most common diseases worldwide. At present, approximately 537 million adults between the ages of 20 and 79 are living with diabetes, corresponding to around 10% of this age group globally (1). Prognoses indicate that this number will increase to 643 million by 2030 and further to 783 million by 2045 (1). As a result, the occurrence of diabetes-related complications is also expected to rise (2). Among individuals with diabetes, diabetic foot ulcers (DFU) develop in approximately 4% -10% and the lifetime risk can be as high as 34% (3). These ulcers frequently become infected, leading to serious complications, significant financial costs, and a markedly increased risk of lower limb amputation (3, 4). In severe cases, infection can progress to sepsis and may even be fatal (3, 4). Xerosis (dry skin) is common among individuals with diabetes (5) and often represents an early entry point of vulnerability for developing foot complications (6).

To minimize the risk of ulcer formation, individuals with diabetes are instructed to inspect their feet daily and to apply foot creams (6). Although topical treatment is fundamental to the management of diabetes-related xerosis, no firm recommendations specify which type of cream should be used, and most commercially available products are neither specifically tailored to nor evaluated in the diabetic population.

Moisturizers are topical formulations intended to improve skin hydration and support barrier function (7, 8). Terminology in this field is not entirely consistent, and the terms *moisturizer* and *emollient* are sometimes used interchangeably because their physicochemical properties and effects can overlap (8). In general, moisturizers are hydrophobic substances that form an occlusive surface film, reducing trans epidermal water loss (TEWL) and helping the skin retain moisture (7, 8). Emollients, on the other hand, are usually non-polar and amphipathic lipids with the ability to soften the skin and improve barrier flexibility through lipid replenishment (7, 8). Several emollients resemble components of the natural stratum corneum lipid matrix and can therefore contribute to barrier repair and improved pliability of the skin (9). Moisturizers and emollients are commonly combined with humectants such as glycerol, urea, lactate, amino acids, and their derivatives (7, 8, 10). Humectants are typically described as substances that attract and retain water within the stratum corneum (7, 8, 10). Beyond their hydrating effects, humectants can also maintain the mobility of proteins and lipids in the stratum corneum under dry conditions, even without necessarily increasing overall hydration levels (11–17). Despite sharing the aim of improving skin hydration, these ingredient classes rely on different but complementary mechanisms (7, 8, 10). However, because most skin care formulations contain multiple ingredients with different and often overlapping mechanisms of action, it is inherently difficult to draw clear categorical boundaries between them. The formulations evaluated in the studies included in this review generally reflected this complexity. Against this background, this review examines studies comparing base formulations with functional creams, defined as formulations containing additional ingredients intended to enhance the performance of the base formulation. In most cases, these additional ingredients are classified as humectants. Accordingly, the categories used in this review should be regarded as pragmatic rather than mechanistic, as many formulations contain multiple potentially active ingredients whose individual contributions cannot readily be distinguished.

Two previous systematic (18, 19) and one scoping (20) review on the topic were identified; none reached clear conclusions, and all highlighted methodological limitations in the studies included (18–20). Importantly, none restricted their inclusion criteria to randomized controlled trials (RCTs). Only one systematic review (18) conducted meta-analyses and assessed risk of bias, but it included non-randomized studies and did not evaluate certainty of evidence. The remaining two reviews presented their findings narratively. Furthermore, the systematic review that conducted meta-analyses focused exclusively on urea-based moisturizer (18), while another review addressed foot xerosis in both diabetic and non-diabetic populations (19). Consequently, the existing evidence base should be interpreted cautiously and cannot support firm clinical recommendations. A new, methodologically rigorous systematic review identifying the most effective ingredients and formulations for topical treatment of diabetic foot xerosis based on evidence from RCTs, preferably conducted in accordance with Cochrane methodological standards (21) is therefore warranted.

## Methods

The methods were pre-specified in a research protocol published in PROSPERO before the review was initiated (registration number: CRD42024579919) (22). There were no language restrictions and no publication period restrictions. The study was conducted in accordance with the Methodological Expectations of Cochrane Intervention Reviews (MECIR) standards (23) and reported in line with the PRISMA 2020 checklist (24) see **Supplementary Table 1** in the **Supplementary Material**.

### Types of studies and participants

We included RCTs that compared different topical treatments for the management of xerosis in people with diabetes with different control interventions, including placebo or vehicle formulations, no treatment, alternative non-humectant formulations, or other topical skin care interventions. Studies were eligible irrespective of language of publication or publication date.

Adults (≥18 years) with type 1 or type 2 diabetes mellitus and xerosis of the feet were included. We excluded animal studies, studies involving participants with foot wounds or ulcers, and studies in which xerosis was attributed to conditions other than diabetes.

### Search methods for identification of studies

A structured search strategy together with an information specialist was developed using controlled vocabulary and free-text keywords related to diabetes, xerosis, and topical formulations. A comprehensive literature search was conducted across several databases, including Medline (EBSCO), Cinahl (EBSCO), Embase, CENTRAL, and Scopus (Elsevier). The latest search date was performed on August 4, 2026. Full search strategies and dates for all databases are available in **Supplementary Tables 2**-**4** in the **Supplementary Material**. This search was supplemented by reference checking and citation searching.

### Data collection

Search results were managed using Covidence systematic review software (Veritas Health Innovation, Melbourne, Australia), which was used for deduplication, screening, and documentation of decisions.

Two reviewers (KB and AE) independently screened titles and abstracts for eligibility. Disagreements were solved by a third reviewer (SA). Two reviewers (KB and PG) then independently assessed full-text reports. Disagreements were resolved through discussion in a group of four (KB, AE, PG and SA).

Two reviewers (KB and SB) independently extracted data from each included study using Covidence data extraction form followed by data entry and management in Cochrane’s Review Manager (RevMan). Disagreements were resolved through discussion or consultation with a third reviewer who also crossed-checked the extracted data to ensure accuracy (SA).

For each included study, we sought all results compatible with each prespecified outcome domain, including all reported measurement instruments, time points, and analyses. Extracted outcomes included: change in xerosis severity (2 and 4 weeks after start of study), skin hydration (corneometry or dermal phase meter, DPM), skin barrier integrity (TEWL), participant satisfaction with cream and adverse events.

In addition, we extracted study-level variables relevant to interpretation and risk of bias. Assumptions made regarding missing, unclear, or ambiguously reported information were explicitly documented and justified in the review records, in accordance with PRISMA 2020 recommendations (24).

We used the Cochrane Risk of Bias tool (RoB 2.0) for randomized controlled studies (25). The assessment was conducted by three reviewers (KB, SB and SA), who worked collaboratively to reach consensus ensuring accurate and balanced judgments.

Two review authors (KB and SA) assessed the certainty of the evidence manually for each important outcome using the GRADE approach (26) Certainty was evaluated across the domains of risk of bias, inconsistency of results, indirectness of evidence, imprecision of effect estimates, large effect and publication bias. For each comparison we prepared a summary of findings table using GRADEpro GDT software.

### Categorization of creams

The categorization of topical formulations was developed after study selection and was not prespecified in the review protocol. It was intended as a pragmatic framework to facilitate a clinically meaningful synthesis of the interventions identified in the included studies. Interventions were grouped at three levels of categorization. First, at the broadest level, a category referred to as *functional cream* was defined as any topical formulation containing at least one added substance intended to improve skin hydration compared with a cream without such a component. Second, a more refined categorization focused on *humectants*, defined as substances intended to enhance skin hydration beyond that achieved by emollient or occlusive ingredients alone. Third, the most specific level of categorization focused on *urea-containing creams*, since the majority of identified and included studies evaluated urea as active intervention. For comparators, all control formulations were pooled into a common category termed *standard base*, defined as a topical cream lacking an additional functional ingredient, against which the intervention was compared. Additionally, studies directly comparing two different humectants were evaluated.

### Statistics

When numerical outcome data were incomplete, unclear, or reported in formats unsuitable for analysis, we contacted study investigators to request clarification or additional information. If the required data could not be obtained, available statistics were converted into analyzable formats using standard methods recommended in Chapter 6 of the Cochrane Handbook for Systematic Reviews of Interventions, such as deriving standard deviations from reported standard errors, confidence intervals, p-values, or interquartile ranges where appropriate (21).

Since included studies assessed xerosis severity using different rating scales and skin hydration were measured at different sites of the foot, treatment effects were summarized using the standardized mean difference (SMD). For each study, the SMD was calculated as the difference between intervention and comparator group means divided by the pooled standard deviation of the outcome among participants. This approach expresses the magnitude of the intervention effect relative to the variability observed within each study and enables results measured using different xerosis severity scales and at different sites of the foot to be combined quantitatively in a meta-analysis (21).

When sufficient data were available and it was appropriate to combine results, we conducted random-effects meta-analyses in RevMan using the inverse-variance method (τ^2^) estimated using the restricted maximum likelihood (REML) method. Confidence intervals for the pooled effect estimates were calculated using the Wald method. Statistical heterogeneity was quantified using the I^2^ statistic.

Sensitivity analyses were conducted to assess the robustness of the primary findings. We repeated the primary analyses after excluding studies judged to be at high risk of bias. Consistency of results across these analyses was interpreted as evidence of robustness.

Meta-analyses included both within-person and parallel-group randomized trials. Sensitivity analyses excluding parallel-group trials were performed to evaluate whether differences in study design influenced the pooled estimates (27).

For studies that used within-person design, effect estimates that accounted for the paired nature of the data were extracted whenever available, in accordance with current Cochrane guidance (27). If paired analyses were not clearly reported, the available published estimates were used. Sensitivity analyses were performed to assess the robustness of the pooled results by excluding studies in which the dependency between paired observations was not clearly accounted for.

### Consumer involvement

As a part of a previous priority-setting work in people with diabetes mellitus and foot care, the research group conducted a study together with participants with diabetes to establish outcomes of this mutual collaboration according to James Lind Alliance methodology (28) to support priority-setting partnerships, where groups of participants with diabetes and clinicians and/or researchers equally defined uncertainties in preventing and treating diabetic foot ulcers (29). Screening/grading skin properties associated with the risk of developing and impaired healing of diabetic foot ulcers (ranked 5) and self-care to prevent diabetic foot ulcer (ranked 7) were uncertainties in this exclusive list of the top 10 most important research priorities.

## Results

### Results of the search

The database searches identified 8386 studies, and two further studies (30, 31) were later identified from citation searching, A total of 49 studies were selected for full-text screening, of which 11 publications met the inclusion criteria, reporting a total of 12 RCTs, as one publication included two RCTs (32) **Table 1**.

**Table 1:** Included studies.

Author	Year	Title
Baker (33)	2008	Effects of a urea-based moisturizer on foot xerosis in people with diabetes
Carter (34)	2013	A study to assess a cosmetic product in the treatment of cracked heels among diabetics
Federici (30)	2012	An urea, arginine and carnosine based cream (Ureadin Rx Db ISDIN) shows greater efficacy in the treatment of severe xerosis of the feet in type 2 diabetic patients in comparison with glycerol-based emollient cream: a randomized, assessor-blinded, controlled trial
Federici (31)	2015	Use of a urea, arginine and carnosine cream versus a standard emollient glycerol cream for treatment of severe xerosis of the feet in patients with type 2 diabetes: a randomized, 8 month, assessor-blinded, controlled trial
Garrigue (35)	2011	Evaluation of the moisturizer Pédimed^®^ in the foot care of diabetic patients
Gin (36)	2017	Treatment by a moisturizer of xerosis and cracks of the feet in individuals with diabetes: a randomized, double-blind, placebo-controlled study
Glonek (37)	2022	Implications of a Diabetic Foot Xerosis Treatment With an Emulsion Containing the Plant-Based Anionic Phospholipids
Martini (38)	2017	Efficacy of an emollient cream in the treatment of xerosis in diabetic foot: a double-blind, randomized, vehicle-controlled clinical trial
Pham (39)	2002	A prospective, randomized, controlled double-blind study of a moisturizer for xerosis of the feet in individuals with diabetes
Quatresooz (32)	2009	Fungal chitin-glucan scaffold for managing diabetic xerosis of the feet in menopausal individuals
Schulte-Walter (40)	2018	Beneficial effects of a topical foam cream product in a study on subjects with type 1 and type 2 diabetic foot skin xerosis

**Figure 1:**
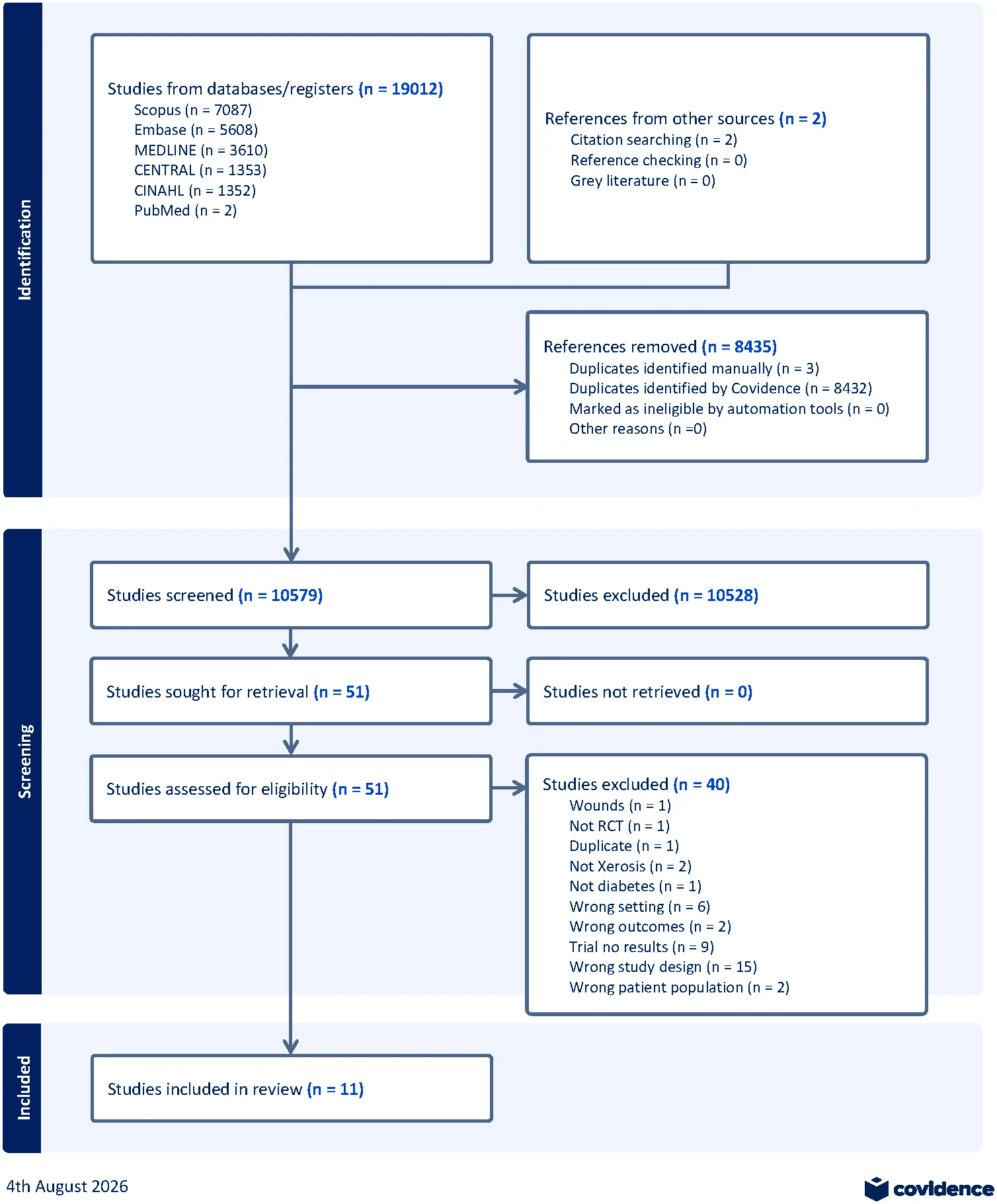
PRISMA flow diagram detailing the study selection process.

To identify any newly published studies, an updated literature search was performed on August 4^th^, 2026. The search was conducted using the same databases, search strategies, and predefined eligibility criteria as the original search. A total of 2189 additional records were retrieved and screened according to the established study selection process. Following title and abstract screening and full-text assessment (n=2), no new studies met the eligibility criteria for inclusion in the review. PRISMA flow diagram detailing the study selection process is presented in **Figure 1**.

Details for all trials regarding the specific formulations, concentrations, control products, and amount of cream applied are presented in **Supplementary Table 5** in the **Supplementary Material**.

Characteristics of participants and studies are reported in **Supplementary Table 6**. Eight of the RCTs were conducted using a double-blinded design (32, 35–40), three were assessor-blinded only (30, 31, 33), and one study provided no information regarding blinding procedures (34). The twelve trials resulted in a total of 603 participants including 949 feet. Nine of the included RCTs (346 participants; 692 feet) used a within-person design meaning that the intervention cream was applied to one foot while the control cream was applied to the other foot simultaneously (32–35, 37–40).

Most studies included similar mean age and diabetes duration. Three of the included studies consisted exclusively of participants with type II diabetes (30, 31, 34), the remaining studies enrolled both type I and type II diabetes.

Across the included studies, most displayed relatively balanced or moderately skewed distributions of men and women. Quattresooz (2009), which consisted of two different RCTs (32), reported an entirely female sample of menopausal women with diabetes and xerotic feet, making it the most specific patient population studied. In contrast, Carter (2013) (34) had a markedly high proportion of men (68%). Two of the studies that did not use a within-person design exhibited imbalanced sex distributions between the intervention and control groups, with one study reporting 32% men in the intervention group compared with 48% in the control group (31), and another reporting 50% versus 30%, respectively (30).

Risk of bias assessments of individual studies and outcomes are presented in the forest plots **Figures 2**–**5**; **Supplementary Figures 1**–**12**. According to Cochrane Risk of Bias tool (RoB 2.0) for randomized controlled studies (25) overall risk of bias was high for two trials (33, 34) and there were some concerns for the remaining ten trials, primarily due to the absence of publicly available study protocols or prespecified analysis plans, resulting in uncertainty regarding potential selective reporting (30–32, 35–40).

**Figure 2:**
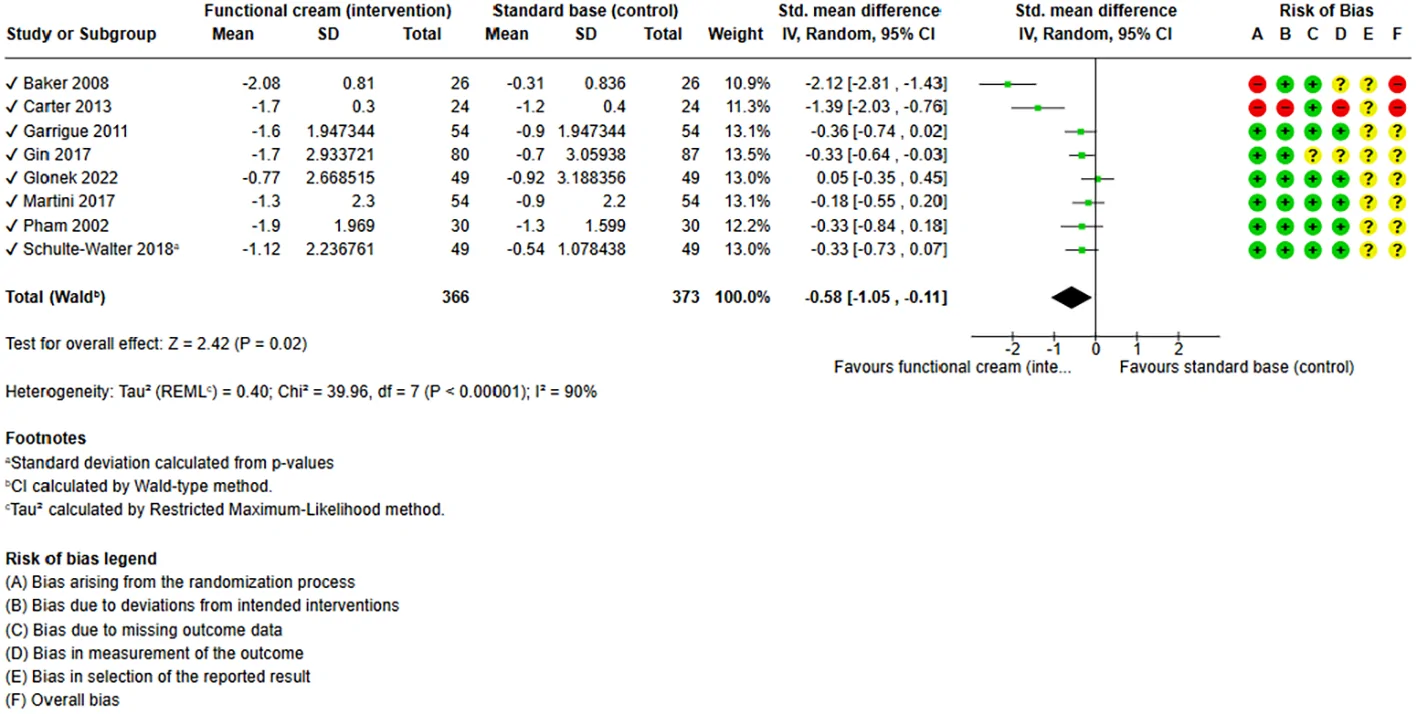
Change from baseline in xerosis grade after two weeks of treatment with a functional cream versus a standard base cream.

**Figure 3:**
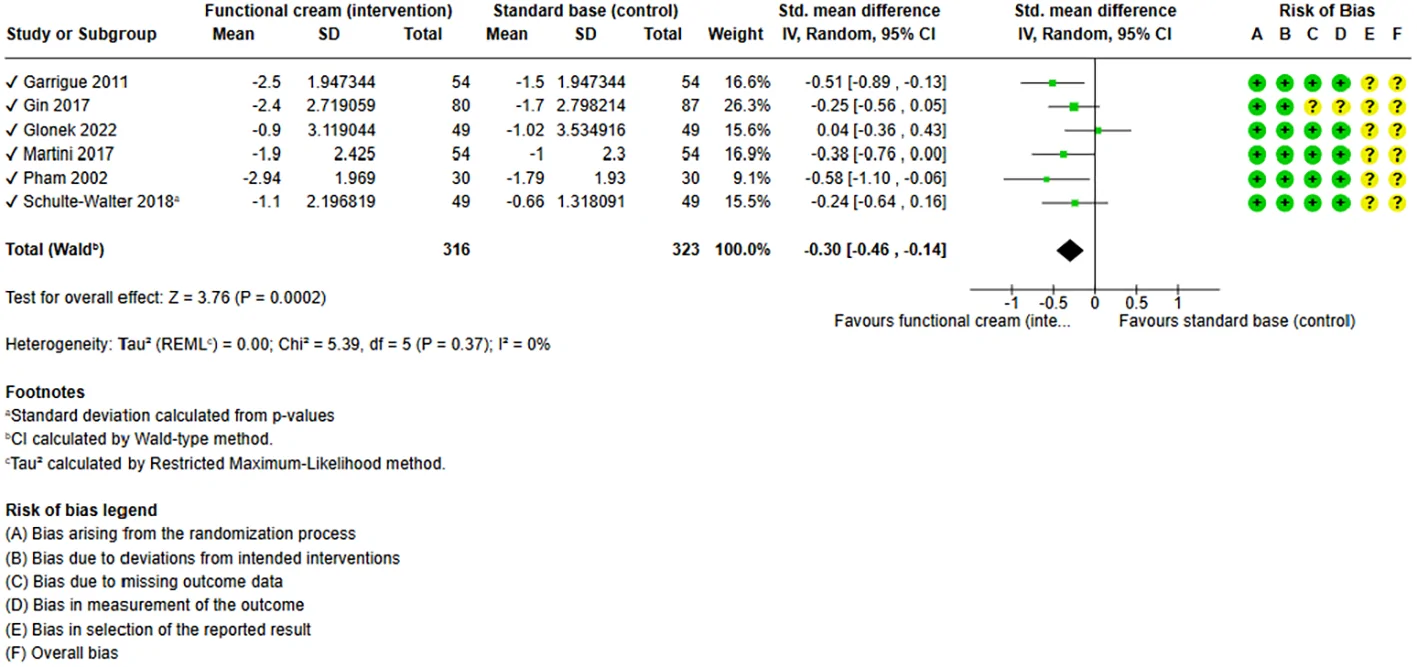
Change from baseline in xerosis grade after four weeks of treatment with a functional cream versus a standard base cream.

**Figure 4:**
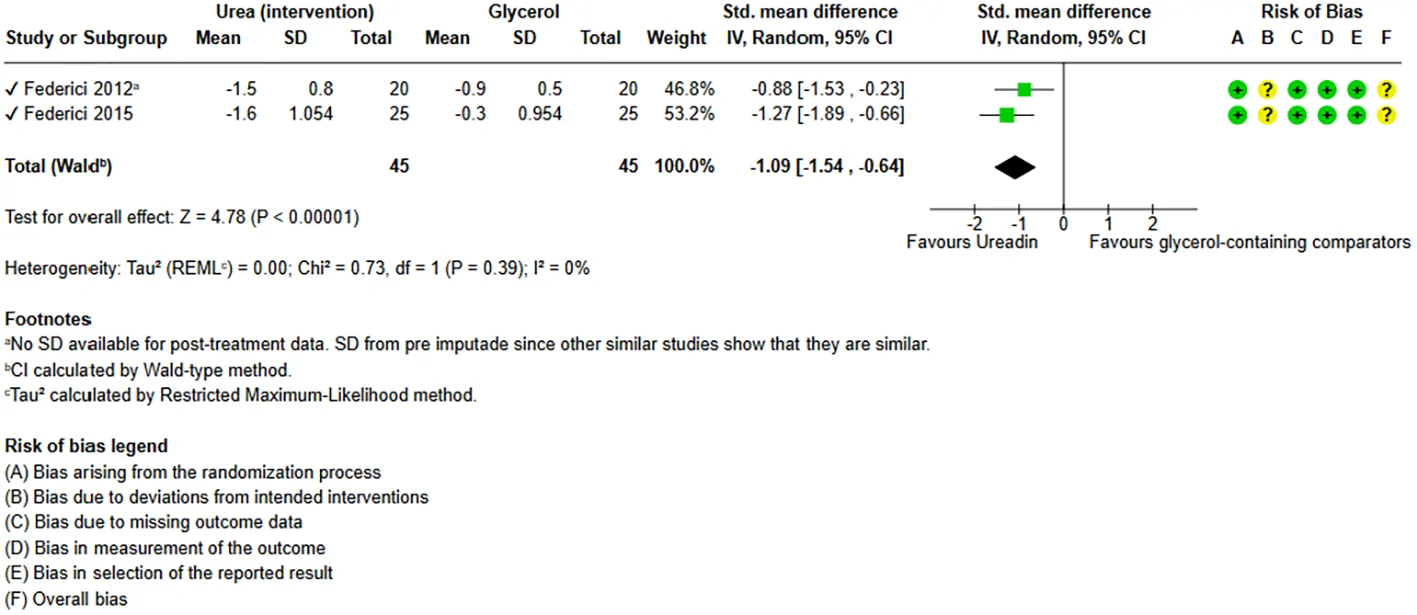
Change from baseline in xerosis grade after four weeks of treatment with Ureadin versus Dexeryl and Neutrogena.

**Figure 5:**
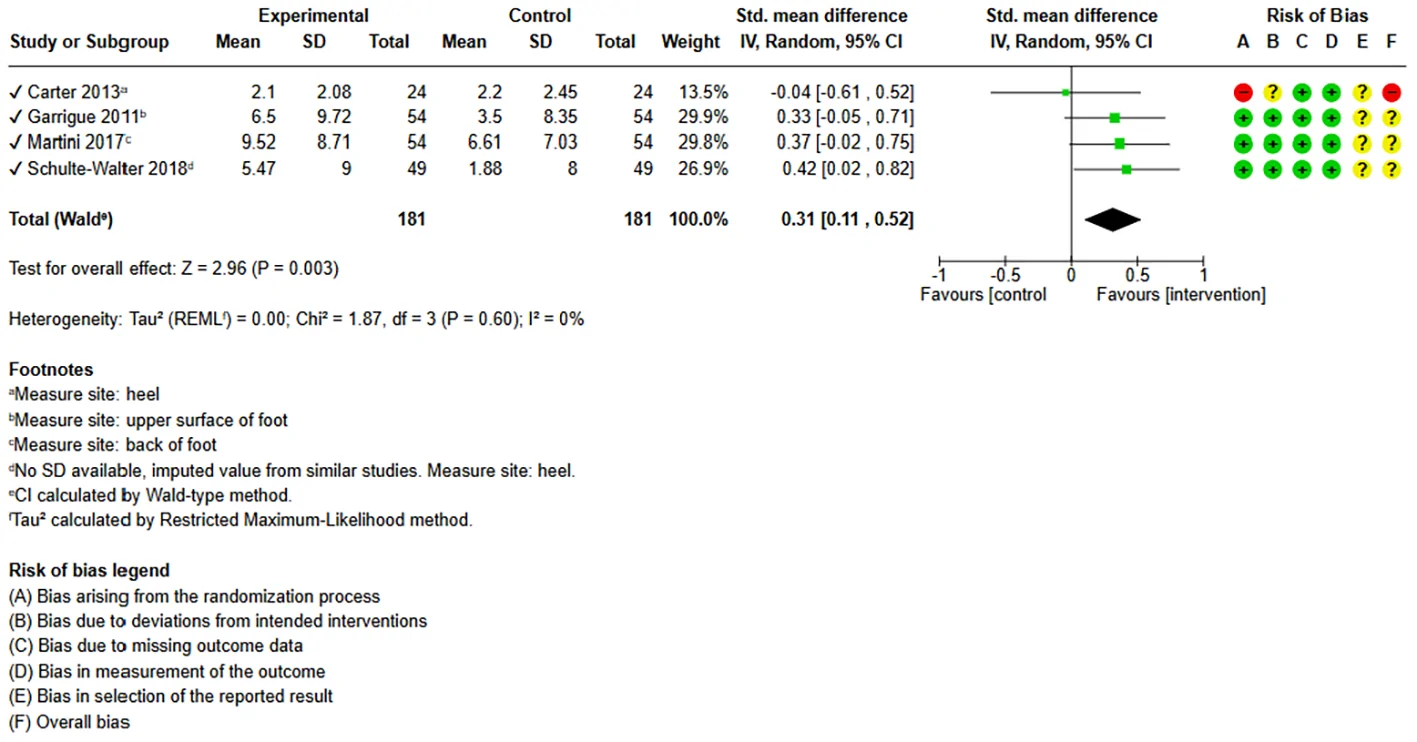
Skin hydration change after two weeks of treatment measured with corneometer functional cream versus standard base.

### Change in foot xerosis severity

Changes in xerosis severity were reported in eight studies after two weeks of treatment with a functional cream compared with a standard base (33–40) and were pooled in a meta-analysis (**Figure 2**). Functional creams were favorable over standard base creams; SMD of -0.58, 95% CI -1.05 to -0.11; p = 0.02). There was statistically significant heterogeneity (I^2^ = 90%, p < 0.00001). A summary of findings table was conducted (**Table 2**). The evidence was of moderate certainty.

**Table 2:** Summary of findings: a functional cream compared to a standard base cream for foot xerosis in participants with diabetes.

Outcomes	Anticipated absolute effects^*^ (95% CI)		Relative effect (95% CI)	№ of participants (studies)	Certainty of the evidence (GRADE)	Comments
	Risk with Standard base (control)	Risk with Functional cream (intervention)				
Change from baseline in xerosis grade after two weeks of treatment assessed with: Visual assessment by trained personnel	-	SMD 0.58 SD lower (1.05 lower to 0.11 lower)	–	453 participants 739 feet (8 RCTs)	⨁⨁⨁◯ Moderate^a,b,c,d,e^	Functional cream (intervention) probably results in a slight reduction in change from baseline in xerosis grade after two weeks of treatment.
Change from baseline in xerosis grade after four weeks of treatment assessed with: Visual assessment by trained personnel	-	SMD 0.3 lower (0.46 lower to 0.14 lower)	–	403 participants 639 feet (6 RCTs)	⨁⨁⨁⨁ High^c,d,f,g^	Functional cream (intervention) results in a slight reduction in change from baseline in xerosis grade after four weeks of treatment.

*The risk in the intervention group (and its 95% confidence interval) is based on the assumed risk in the comparison group and the relative effect of the intervention (and its 95% CI).

CI, confidence interval; SMD, standardized mean difference.

GRADE Working Group grades of evidence.

High certainty: we are very confident that the true effect lies close to that of the estimate of the effect.

Moderate certainty: we are moderately confident in the effect estimate: the true effect is likely to be close to the estimate of the effect, but there is a possibility that it is substantially different.

Low certainty: our confidence in the effect estimate is limited: the true effect may be substantially different from the estimate of the effect.

Very low certainty: we have very little confidence in the effect estimate: the true effect is likely to be substantially different from the estimate of effect.

^a^ One study (Carter et al.) had a high risk of bias but contributed little weight. All studies showed some concern for selective reporting only because no protocols were available, which is expected given their remote publication date. We found no indication of actual selective reporting, so we judged this as not a serious concern.

^b^ Two studies (Baker et al. and Carter et al.) are the reasons for the inconsistency and high heterogeneity but they have low weights and point in the same direction why we judge as serious and not very serious concerns.

^c^ Study design, population, and outcomes are directly applicable to the clinical question; there is no reason for downgrading.

^d^ The results are based on sufficiently large studies with narrow confidence intervals and clear effects.

^e^ We only included RCTs. Only observational studies are evaluated in this domain.

^f^ Most studies show a low risk of bias, and any potential limitations are considered minor and not relevant to the outcomes.

^g^ The results are consistent across studies with similar designs and populations.

Seven of these investigations assessed a humectant-based formulation (33–36, 38–40) (**Supplementary Figure 1**, **Supplementary Table 7**) and six specifically evaluated urea as the active component (33–36, 39, 40) (**Supplementary Figure 2**, **Supplementary Table 8**). The functional creams were favorable over standard base creams with statistically significant heterogeneity.

To assess the robustness of the primary findings, a sensitivity analysis was performed in which the two studies judged to have a high risk of bias (33, 34) were excluded from the pooled analysis (**Supplementary Figures 3**-**5**). After removal of these studies, the direction of effect remained unchanged, and the magnitude of xerosis reduction continued to favor the intervention over the standard base. For functional cream versus standard base, the pooled effect estimate remained significant (SMD -0.25, 95% CI -0.41 to -0.09; p = 0.002), with no statistically significant heterogeneity (I^2^ = 0%, p = 0.67) (**Supplementary Figure 3**).

A sensitivity analysis excluding the study that used parallel-group design (36) was performed to evaluate whether differences in study design influenced the pooled estimates (**Supplementary Figure 6**). The SMD remained virtually unaffected.

A sensitivity analysis was performed by excluding three studies (38–40) for which it was unclear whether the dependency between paired observations had been fully accounted for (**Supplementary Figure 7**). After removal of these studies, the magnitude of xerosis reduction continued to favor the intervention over the standard base (SMD -0.79, 95% CI -1.55 to -0.03; p = 0.04).

After four weeks of treatment, six studies reported changes from baseline in xerosis grade (35–40). The functional creams were favorable over standard base cream, with an SMD of -0.30, 95% CI -0.46 to -0.14; p = 0.0002), without heterogeneity (I^2^ = 0%) *(***Figure 3**, **Table 2**). The evidence was of high certainty.

Five of these assessed humectant-based formulations (35, 36, 38–40) (**Supplementary Figure 8**, **Supplementary Table 7**), and four included urea-based interventions (35, 36, 39, 40) (**Supplementary Figure 9**, **Supplementary Table 8**). Creams containing urea were more effective than standard base creams after four weeks (SMD -0.36, 95% CI -0.55 to - 0.17), with high-certainty evidence.

Two studies evaluated two humectant formulations against each other, containing either urea or glycerol. One study compared Ureadin vs Dexeryl (30) and another Ureadin vs Neutrogena (31), assessing reductions in xerosis after four weeks of treatment (see **Figure 4** and **Table 3**). In both trials, Ureadin demonstrated superior efficacy, showing consistently greater reductions in xerosis severity than the glycerol-containing comparators (SMD -1.09, 95% CI -1.54 to -0.64; p< 0.00001; I^2^ = 0%). These results are presented separately due to the different comparator structure. The certainty of evidence was high.

**Table 3:** Summary of findings: Ureadin compared to Dexeryl and Neutrogena for foot xerosis in participants with diabetes.

Outcomes	Anticipated absolute effects^*^ (95% CI)		Relative effect (95% CI)	№ of participants (studies)	Certainty of the evidence (GRADE)	Comments
	Risk with Dexeryl	Risk with Ureadin (intervention)				
Change from baseline in xerosis grade after four weeks of treatment	-	SMD 1.09 lower (1.54 lower to 0.64 lower)	–	90 participants 90 feet (2 RCTs)	⨁⨁⨁⨁ High^a,b,c,d,e^	Ureadin (intervention) results in a reduction in change from baseline in xerosis grade after four weeks of treatment.

*The risk in the intervention group (and its 95% confidence interval) is based on the assumed risk in the comparison group and the relative effect of the intervention (and its 95% CI).

CI, confidence interval; SMD, standardized mean difference.

GRADE Working Group grades of evidence.

High certainty: we are very confident that the true effect lies close to that of the estimate of the effect.

Moderate certainty: we are moderately confident in the effect estimate: the true effect is likely to be close to the estimate of the effect, but there is a possibility that it is substantially different.

Low certainty: our confidence in the effect estimate is limited: the true effect may be substantially different from the estimate of the effect.

Very low certainty: we have very little confidence in the effect estimate: the true effect is likely to be substantially different from the estimate of effect.

^a^ Only assessor blinded studies which leads to some concerns but no suspicion of serious risk of bias.

^b^ Study design, population, and outcomes are directly applicable to the clinical question; there is no reason for downgrading.

^c^ The results are based on sufficiently large studies with narrow confidence intervals and clear effects.

^d^ We only included RCTs. Only observational studies are evaluated in this domain.

^e^ The results are consistent across studies with similar designs and populations.

### Change in skin hydration

Four studies reported changes in skin hydration measured by corneometry after two weeks of treatment, comparing a functional cream with a standard base (34, 35, 38, 40). Greater improvements in skin hydration were shown in the group treated with the functional cream (SMD 0.31, 95% CI 0.11 to 0.52; p = 0.003; I ^2^ = 0%) (**Figure 5**).

Three of the studies also assessed skin hydration after four weeks of treatment (35, 38, 40), and the beneficial effects were sustained (SMD 0.43, 95% CI 0.20 to 0.65; p = 0.0002) (**Supplementary Figure 10**). Certainty of the evidence was low after both two and four weeks of treatment. A detailed summary of the certainty of evidence and the magnitude of effects is presented in the summary of findings (**Table 4**). 

**Table 4:** Summary of findings: Functional cream (intervention) compared to Standard base (control) for improved skin hydration in participants with diabetes.

Outcomes	Anticipated absolute effects^*^ (95% CI)		Relative effect (95% CI)	№ of participants (studies)	Certainty of the evidence (GRADE)	Comments
	Risk with Standard base (control)	Risk with Functional cream (intervention)				
Skin hydration change after two weeks of treatment Functional cream versus Standard base assessed with: Corneometer	-	SMD 0.31 higher (0.11 higher to 0.52 higher)	–	181 participants 362 feet (4 RCTs)	⨁⨁◯◯ Low^a,b,c^	The evidence suggests functional cream (intervention) results in a slight increase in skin hydration change after two weeks of treatment.
Skin hydration change after four weeks of treatment Functional cream versus Standard base assessed with: Corneometer	-	SMD 0.43 higher (0.2 higher to 0.65 higher)	–	157 participants 314 feet (3 RCTs)	⨁⨁◯◯ Low^b,c^	The evidence suggests functional cream (intervention) results in a slight increase in skin hydration change after four weeks of treatment.
Skin hydration change after two weeks Functional cream versus Standard base assessed with: Dermal Phase Meter (DPM)	-	SMD 1.8 higher (1.09 higher to 2.5 higher)	–	30 participants 180 sites (1 RCT)	⨁◯◯◯ Very low^b,c,d^	Functional cream (intervention) may increase skin hydration after two weeks but the evidence is very uncertain.
Skin hydration change after two weeks Humectant vs Humectant assessed with: Dermal Phase Meter (DPM)	-	SMD 0.41 higher (0.12 higher to 0.71 higher)	–	30 participants 180 sites (1 RCT)	⨁◯◯◯ Very low^b,c,d^	Intervention may increase skin hydration after two weeks but the evidence is very uncertain.^d^

*The risk in the intervention group (and its 95% confidence interval) is based on the assumed risk in the comparison group and the relative effect of the intervention (and its 95% CI).

CI, confidence interval; SMD, standardized mean difference.

GRADE Working Group grades of evidence.

High certainty: we are very confident that the true effect lies close to that of the estimate of the effect.

Moderate certainty: we are moderately confident in the effect estimate: the true effect is likely to be close to the estimate of the effect, but there is a possibility that it is substantially different.

Low certainty: our confidence in the effect estimate is limited: the true effect may be substantially different from the estimate of the effect.

Very low certainty: we have very little confidence in the effect estimate: the true effect is likely to be substantially different from the estimate of effect.

Explanations.

^a^ One study (Carter et al.) had a high risk of bias but contributed little weight. All studies showed some concern for selective reporting only because no protocols were available, which is expected given their remote publication date. We found no indication of actual selective reporting, so we judged this as not a serious concern.

^b^ Measurements at different sites of the foot.

^c^ Wide confidence intervals, large standard deviations, small patient populations.

^d^ Patient population consists only of menopausal women with diabetes mellitus.

In the within-person design study that comprised two separate RCTs (32), skin hydration was measured using a dermal phase meter (DPM) at three anatomical sites on each foot (toe, heel, and vault). These site-specific measurements were not combined, as no participant-level summary values were reported. Instead, each anatomical site was treated as a separate outcome, while recognizing that the measurements were correlated because they were obtained from the same participants. The first trial (RCT A) assessed a functional cream with a standard base after two weeks of treatment. The second trial (RCT B) evaluated the same outcome over the same duration but compared two different humectant formulations, see **Supplementary Table 5**. Findings from both trials are presented in **Supplementary Figures 11**, **12**; **Table 4**. The forest plots and summary of findings table should primarily be regarded as a visual summary of the available evidence rather than a precise quantitative estimate of treatment effect. Owing to heterogeneity in anatomical locations for the measurements and that they derived from the same 30 participants, the pooled estimate has limited interpretability. Nevertheless, the individual measurements estimate consistently favored the intervention across the evaluated anatomical locations, supporting a narrative interpretation of an overall beneficial treatment effect. The certainty of evidence was very low for both RCT A and RCT B.

### Change in skin barrier integrity

TEWL reflects passive water diffusion from the body through the stratum corneum and indicates barrier integrity. One study (40) measured changes in TEWL using an AquaFlux™ (Model AF200, Biox Systems Ltd, London, England). The intervention demonstrated a statistically significant superiority over control at the two-week timepoint, reflected by a greater reduction in TEWL from baseline (p = 0.029). This difference was not statistically significant at the end of the study after four weeks (p = 0.088) (**Table 5**).

**Table 5:** TEWL-change from unknown baseline in study (39).

Timepoints	Intervention Allpresan foam cream B, active Mean	Control Allpresan foam cream A, vehicle Mean	p-value
Halftime (2 weeks)	-2.50	-0.54	0.029
End of study (4 weeks)	-4.35	-2.65	0.088

### Adverse events and participation satisfaction

Nine RCTs reported on adverse events (31–36, 38, 39). One study reported five adverse events (35), four were unrelated, and one mild burning sensation was non-assessable. Across the remaining studies, adverse events were reported in only two trials. In Gin (2017) (36), three events occurred in the intervention group and one in the control; all were mild/moderate and resolved without discontinuation. In Martini (2017) (38), one intervention-group case (interdigital maceration and cracking) was judged as probably treatment-related.

Participants’ satisfaction with the creams was reported as an outcome in five studies (33, 34, 37, 38, 40). Although this represents an important and clinically relevant measure, the heterogeneity in reporting methods made it difficult to synthesize the data.

## Discussion

### Summary of main results and limitations of the evidence

This systematic review assessed evidence from 12 RCTs comparing formulations of topical treatments for primary foot xerosis in individuals with diabetes mellitus type 1 or type 2. The included studies encompassed a diverse range of topical formulations, with variation in the type and concentration of functional ingredients, as well as differences in vehicle composition. Many formulations contained multiple active ingredients with overlapping or potentially synergistic functions (**Supplementary Table 5**), making it difficult to attribute observed effects to individual components or determine the relative contribution of specific ingredients. In addition, differences in treatment regimens, including treatment duration and application frequency, may have influenced the magnitude of observed effects. Beyond formulation-related factors, clinical and methodological heterogeneity was introduced by differences in comparator formulations, and outcome assessment methods. Nine studies had a within-person trial design, and three studies had a parallel group trial design. These factors complicate direct comparisons between studies and should be considered when interpreting the pooled estimates and the overall evidence base.

A particularly important source of outcome-related heterogeneity was the substantial variation in xerosis severity scales used across the included studies. Although all studies aimed to assess the same clinical outcome, different scoring systems were applied, limiting direct comparison of treatment effects and reducing the clinical interpretability of pooled estimates. Six studies (31, 34–36, 38, 39) used the Xerosis Assessment Scale (XAS), ranging from 0 to 8, whereas other studies applied scales ranging from 1–5 (33), 0–4 (30), 0–3 (37), and 1–3 (40). Consequently, SMDs, rather than mean differences (MDs), were used in the meta-analyses to enable pooling across different measurement instruments. While the SMD approach facilitates statistical synthesis of outcomes assessed using different scales, it expresses treatment effects in standard deviation units rather than on a clinically intuitive scale, making the magnitude of the effect more difficult to interpret in clinical practice.

Despite these challenges, the overall findings consistently indicated that functional creams appear to be more effective than standard base formulations in improving xerosis-related outcomes (**Figures 2**, **4**, **5**; **Supplementary Figures 3, 6, 7, 10, 11**; **Tables 2**, **4**). This suggests that formulations containing targeted, active components may offer additional clinical benefits beyond that of basic emollient therapy, even if the precise mechanisms and relative contributions of individual ingredients remain difficult to disentangle.

After excluding the two studies assessed as having a high risk of bias (33, 34), the sensitivity analysis (**Supplementary Figure 3**) showed that the pooled effect remained statistically significant, although the magnitude of the effect was reduced, while heterogeneity decreased considerably (from I^2^ 90% to I^2^ 0%) compared to the primary analysis (**Figure 2**). Both Baker (33) and Carter (34) reported larger treatment effects, which may be partly explained by methodological limitations. In Carter (34), the intervention was administered to participants with more severe baseline foot xerosis, allowing greater scope for improvement and thereby potentially inflating the observed effect size. This imbalance was also a key reason for the high risk of bias assessment. In Baker (33), the control consisted of participants'; usual creams, encompassing four different formulations (**Supplementary Table 5**), which introduced heterogeneity and reduced the reliability of the comparison. The remaining studies were assessed as having some concerns regarding risk of bias, primarily because no published study protocols were available, raising the possibility of selective reporting. However, prospective protocol registration was not common practice when most of these studies were conducted and published, which may be considered a mitigating factor.

The exclusion of Baker and Carter (33, 34) also enabled a comparison of treatment effects across timepoints, as all remaining studies assessed outcomes at both two and four weeks of treatment (**Figure 3**; **Supplementary Figure 3**). The pooled effect estimate was slightly greater after four weeks of treatment compared with two weeks.

In the more refined categorization comparing humectant-based formulations with standard base creams, only one study, Glonek (37), evaluated an intervention that could not be classified as a humectant. In this study, a plant-based anionic polar phospholipid (APP) formulation, acting primarily as an emollient, was compared with an occlusive moisturizer based on mineral oil hydrocarbons. This study also demonstrated the smallest effect size in the forest plot (**Figures 2**, **3**). Given that this observation is based on a single study, no firm conclusions can be drawn regarding comparative effectiveness between humectant and non-humectant formulations, such as APP.

Urea was the most frequently studied ingredient, likely reflecting greater research focus. Consequently, the evidence base for urea is larger than for other ingredients, which should be considered when interpreting the apparent strength of evidence. In the second, more refined categorization focusing on humectant formulations, six out of seven studies compared urea with a standard base treatment (33–36, 39, 40), whereas one study (38) compared Dexeryl, a glycerol-containing formulation, with its vehicle lacking glycerol (See **Supplementary Table 5**). Notably, this study demonstrated the smallest effect size in the forest plot after two weeks of treatment (**Supplementary Figure 1**). Although this observation is based on a single study and should be interpreted with caution, it may tentatively suggest that urea-based formulations are associated with greater treatment effects compared with glycerol. This interpretation is further supported by the two studies (30, 31) that directly compare a urea-based formulation (Ureadin) with glycerol-based formulations - Neutrogena (40% glycerol) (31) and Dexeryl (15% glycerol) (30) see **Figure 3**, **Table 4**.

Although the meta-analysis examined “urea-based” treatments (**Figure 3**, **Supplementary Figures 2, 5, 9**), substantial heterogeneity existed across studies in urea concentrations (5–25%), and accompanying ingredients (see **Supplementary Table 5**). Consequently, the pooled analysis reflects a heterogeneous group of formulations rather than a single uniform intervention, precluding assessment of a dose-response relationship and limiting the ability to attribute the observed effects exclusively to urea or to specific urea concentrations. These observations may be interpreted considering the known effects of key humectants such as glycerol, urea, and propylene glycol. These substances share similar mechanisms of action, including the ability to maintain molecular mobility within the stratum corneum under dry conditions, which may contribute to preserved barrier function even in the absence of a measurable increase in water content *in vitro* (11, 41). Clinically, however, their use has consistently been associated with improved hydration scores, suggesting an overall beneficial effect on skin hydration and xerosis (42, 43). Taken together with the comparative findings, this may help explain why formulations containing humectants (particularly urea-based products) tend to demonstrate greater efficacy than formulations lacking these components.

The findings related to skin hydration indicated that functional creams provide a modest but consistent improvement compared with standard base formulations (**Figure 5**; **Supplementary Figures 10**, **11**; **Table 4**). After two weeks of treatment, a small but statistically significant effect was observed (**Figure 5**), which appeared to increase slightly after four weeks (**Supplementary Figure 10**), suggesting a sustained and potentially cumulative benefit over time. However, the certainty of the evidence was rated as low at both timepoints, which limits the strength of the conclusions that can be drawn. Similarly, the study assessing skin hydration with DPM (32) demonstrated improved outcomes with functional creams, although the certainty of evidence was rated as very low (**Supplementary Figures 11**, **12**; **Table 4**). The very low certainty reflects several factors, including measurements taken at different sites of the foot, wide confidence intervals, large standard deviations, small sample sizes, and restriction of the study population consisting to menopausal women with diabetes mellitus (32).

One study (40), evaluated skin barrier integrity using TEWL, providing limited but suggestive evidence of a beneficial effect of functional creams (**Table 5**). A statistically significant reduction in TEWL was observed after two weeks of treatment, indicating improved skin barrier integrity compared with the control. However, this effect was not sustained for four weeks, at which point the difference between groups was no longer statistically significant. Interpretation of these results is limited by reliance on a single study, lack of reported standard deviations precluding assessment of precision, and unclear baseline data. Consequently, the effects of functional creams on skin barrier integrity, assessed by TEWL, remain uncertain and require confirmation in more rigorously reported studies.

Few adverse events were reported, although the limited sample sizes and short follow-up periods preclude definitive conclusions regarding comparative safety. Participants’ satisfaction with the creams was reported as an outcome in five studies (33, 34, 37, 38, 40). Although this represents an important and clinically relevant measure, the heterogeneity in reporting methods made it difficult to synthesize the data. The absence of standardized assessment tools limits comparability, highlighting the need for more consistent and validated approaches in future studies.

Overall, the included RCTs reported broadly similar participant characteristics in terms of age and diabetes duration, although some studies lacked complete reporting. The distribution of sex was generally balanced across studies, although some trials reported uneven sex distributions between intervention and control groups, and one study consisted of only menopausal women (32). Participants mainly comprised mixed populations with type I and type II diabetes, with a small number of studies focusing on only diabetes type II. Most trials applied double-blinded procedures.

Nine of the included RCTs used a within-person design (**Supplementary Table 6**). This design offers several methodological advantages, most notably the reduction of inter-individual variability, minimizing the influence of confounding factors. As a result, treatment effects can often be detected with greater precision even in relatively small sample sizes (44, 45). However, within-person designs also have limitations. Since both feet belong to the same individual, there is a potential risk for cross-contamination, especially when treatments are self-administered. Additionally, feet may not be symmetrical at baseline, and differences between the right and left foot may introduce meaningful asymmetries. Such designs may also increase the risk of unblinding, due to direct within-participant comparison of local effects. This risk may be further influenced by participants recognizing products from prior use or by noticeable differences in smell, color, or consistency between formulations. Therefore, the use of objective outcome measures is recommended whenever possible (44–46).

Another methodological challenge is the inclusion of both within-person and parallel-group randomized trials in the same meta-analyses, although current methodological guidance supports combining these designs when appropriate (27). However, the reporting of statistical methods and effect estimates was insufficient in a small number of studies (38–40), making it difficult to determine whether within-person dependency had been fully accounted for. Exclusion of the parallel-group trial (**Supplementary Figure 6**) did not materially change the pooled estimates, supporting the robustness of the findings across study designs.

Assessment of publication bias in the present systematic review was not justified, as no more than eight studies were included in any meta-analysis. Tests for funnel plot asymmetry are generally recommended only when at least ten studies are available, because their statistical power is low with fewer studies (21). However, small-study effects cannot be completely excluded and may represent an additional source of uncertainty when interpreting the pooled estimates. Small studies with null results are less likely published, whereas small trials, on the other hand, may systematically inflate effect size estimates compared to larger trials.

### Agreements and disagreements with other studies or reviews

The findings of this systematic review with meta-analysis of RCTs are broadly consistent with previous reviews (18–20), which have highlighted substantial heterogeneity in individual studies in study design, intervention, controls, outcome measures, and overall methodological quality, limiting the ability to draw firm conclusions regarding the superiority of specific active ingredients. Like earlier work, urea emerged as one of the most frequently studied components (18–20). This review is also in line with a previous report suggesting that regular use of moisturizers, particularly those containing urea or glycerol, can improve dry skin in individuals with diabetes (20). Results support earlier observations that humectant-containing formulations may be particularly beneficial in this population. However, as highlighted in previous literature, important gaps remain regarding optimal treatment regimens, including frequency and duration of use, as well as the long-term clinical relevance of these interventions (20). In contrast to previous reviews, which were limited by heterogeneous study designs (18–20), inclusion of non-randomized evidence (18–20), and lack of certainty assessments (18–20), this review applies more rigorous methodological standards by restricting inclusion to RCTs and systematically evaluating risk of bias and certainty of evidence. As a result, the findings of this updated review provide a more robust and reliable synthesis of the available evidence. Although limitations remain, this approach strengthens confidence in the overall conclusions and offers a more solid basis for interpreting the effectiveness of topical treatments for diabetic foot xerosis.

### Implications for research

Future research should prioritize well-designed, adequately powered RCTs with longer follow-up periods to evaluate the sustained effects of topical interventions. While the included studies demonstrated short-term improvements in outcomes such as reduced foot xerosis severity, improved skin hydration and barrier integrity, the clinical relevance of these changes remains uncertain. It is important to determine whether such improvements translate into meaningful long-term outcomes, including a reduced risk of foot ulceration, amputation, or mortality. In addition, future studies should aim for more standardized intervention and comparator formulations, to enable more precise comparisons between treatments. Improved methodological rigor, including prospective protocol registration and comprehensive reporting of outcomes would further strengthen the evidence base. Reporting standards for foot xerosis treatments need to be developed, including agreement on a standardized scale for assessing xerosis severity. Since urea was the most frequently studied ingredient, the stronger evidence available for urea likely reflects greater research focus rather than proven superiority. Further well-designed studies evaluating other humectants are warranted to determine whether similar effects can be achieved with alternative formulations.

## Conclusions

In conclusion, current evidence suggests that topical treatments reduce foot xerosis in individuals with diabetes, with functional creams, particularly those containing humectants, demonstrating greater efficacy than standard base formulations. Among humectants, urea-based formulations are the most extensively studied and show consistent benefits. However, due to heterogeneity in formulations, and outcome measures, it remains difficult to determine the superiority of specific active ingredients. Therefore, current evidence does not support definitive recommendations favoring urea over other humectant-containing formulations. Reporting standards in topical treatment of foot xerosis need to be developed, and further high-quality studies with clinically relevant long-term outcomes such as occurrence of foot ulcers are needed to strengthen the evidence base and guide clinical practice.

## Data Availability

The data analyzed in this study is subject to the following licenses/restrictions: This is a systematic review based on published data and we have created datasets in RevMan. Requests to access these datasets should be directed to karin.borgstrom@mau.se.
