# Supplementary material for: Moisturizer formulations for diabetic foot xerosis: a systematic review and meta-analysis of randomized controlled trials
Source: Front Clin Diabetes Healthc. 2026 Sep 3;7:1922860. doi: 10.3389/fcdhc.2026.1922860 (PMC13581737; doi:10.3389/fcdhc.2026.1922860)
Supplement: Supplementary file 1 [file SupplementaryFile1.pdf]

Supplementary Material

Supplementary Table 1. PRISMA check list

Section and Topic	Item #	Checklist item	Location where item is reported
**TITLE**			
Title	1	Identify the report as a systematic review.	Title
**ABSTRACT**			
Abstract	2	See the PRISMA 2020 for Abstracts checklist.	Page 2
**INTRODUCTION**			
Rationale	3	Describe the rationale for the review in the context of existing knowledge.	Page 3 Last paragraph Introduction
Objectives	4	Provide an explicit statement of the objective(s) or question(s) the review addresses.	Page 3 Last paragraph Introduction
**METHODS**			
Eligibility criteria	5	Specify the inclusion and exclusion criteria for the review and how studies were grouped for the syntheses.	Page 3 "Types of studies and participants" Page 4 "Categorisation of creams"
Information sources	6	Specify all databases, registers, websites, organisations, reference lists and other sources searched or consulted to identify studies. Specify the date when each source was last searched or consulted.	Page 3-4 "Search methods for identification of studies"
Search strategy	7	Present the full search strategies for all databases, registers and websites, including any filters and limits used.	Appendix Supplementary Tables 2-4
Selection process	8	Specify the methods used to decide whether a study met the inclusion criteria of the review, including how many reviewers screened each record and each report retrieved, whether they worked independently, and if applicable, details of automation tools used in the process.	Page 4 "Data collection"

Section and Topic	Item #	Checklist item	Location where item is reported
Data collection process	9	Specify the methods used to collect data from reports, including how many reviewers collected data from each report, whether they worked independently, any processes for obtaining or confirming data from study investigators, and if applicable, details of automation tools used in the process.	Page 4 “Data collection”
Data items	10a	List and define all outcomes for which data were sought. Specify whether all results that were compatible with each outcome domain in each study were sought (e.g. for all measures, time points, analyses), and if not, the methods used to decide which results to collect.	Page 4 “Data collection”
	10b	List and define all other variables for which data were sought (e.g. participant and intervention characteristics, funding sources). Describe any assumptions made about any missing or unclear information.	Page 4 “Data collection and Page 5 “Statistics””
Study risk of bias assessment	11	Specify the methods used to assess risk of bias in the included studies, including details of the tool(s) used, how many reviewers assessed each study and whether they worked independently, and if applicable, details of automation tools used in the process.	Page 4 “Data collection”
Effect measures	12	Specify for each outcome the effect measure(s) (e.g. risk ratio, mean difference) used in the synthesis or presentation of results.	Page 5 “Statistics”
Synthesis methods	13a	Describe the processes used to decide which studies were eligible for each synthesis (e.g. tabulating the study intervention characteristics and comparing against the planned groups for each synthesis (item #5)).	Supplementary Table 5<br>Page 4 “Categorisation of creams”<br>Page 4 “Data collection”<br>Page 6 “Statistics”
	13b	Describe any methods required to prepare the data for presentation or synthesis, such as handling of missing summary statistics, or data conversions.	Page 5 “Statitics”
	13c	Describe any methods used to tabulate or visually display results of individual studies and syntheses.	Supplementary Table 5<br>Page 4 “Categorisation of creams”<br>Page 4 “Data collection”<br>Page 5 “Statistics”
	13d	Describe any methods used to synthesize results and provide a rationale for the choice(s). If meta-analysis was performed, describe the model(s), method(s) to identify the presence and extent of statistical heterogeneity, and software package(s) used.	Page 4 “Categorisation

Section and Topic	Item #	Checklist item	Location where item is reported
			of creams" Page 5 "Statistics"
	13e	Describe any methods used to explore possible causes of heterogeneity among study results (e.g. subgroup analysis, meta-regression).	Page 5 "Statistics"
	13f	Describe any sensitivity analyses conducted to assess robustness of the synthesized results.	Page 5 "Statistics"
Reporting bias assessment	14	Describe any methods used to assess risk of bias due to missing results in a synthesis (arising from reporting biases).	Page 4 "Data collection"
Certainty assessment	15	Describe any methods used to assess certainty (or confidence) in the body of evidence for an outcome.	Page 4 "Data collection"
**RESULTS**			
S7-S8Study selection	16a	Describe the results of the search and selection process, from the number of records identified in the search to the number of studies included in the review, ideally using a flow diagram.	Figure 1<br>Page 5 and 6 "Results of the search"
	16b	Cite studies that might appear to meet the inclusion criteria, but which were excluded, and explain why they were excluded.	Figure 1
Study characteristics	17	Cite each included study and present its characteristics.	Table 1<br>Supplementary Tables 5-6
Risk of bias in studies	18	Present assessments of risk of bias for each included study.	Figure 2-5<br>Supplementary Figures 1-12
Results of individual studies	19	For all outcomes, present, for each study: (a) summary statistics for each group (where appropriate) and (b) an effect estimate and its precision (e.g. confidence/credible interval), ideally using structured tables or plots.	Table 2-5<br>Supplementary Tables 6-8<br>Figure 2-5<br>Supplementary Figures 1-12
Results of syntheses	20a	For each synthesis, briefly summarise the characteristics and risk of bias among contributing studies.	Table 2-5<br>Supplementary Tables 5-8

Section and Topic	Item #	Checklist item	Location where item is reported
			Figure 2-5<br>Supplementary Figures 1-12
	20b	Present results of all statistical syntheses conducted. If meta-analysis was done, present for each the summary estimate and its precision (e.g. confidence/credible interval) and measures of statistical heterogeneity. If comparing groups, describe the direction of the effect.	Table 2-5<br>Supplementary Tables 6-8<br>Figure 2-5<br>Supplementary Figures 1-12
	20c	Present results of all investigations of possible causes of heterogeneity among study results.	Supplementary Figures 3-5
	20d	Present results of all sensitivity analyses conducted to assess the robustness of the synthesized results.	Supplementary Figures 3-7
Reporting biases	21	Present assessments of risk of bias due to missing results (arising from reporting biases) for each synthesis assessed.	N.A
Certainty of evidence	22	Present assessments of certainty (or confidence) in the body of evidence for each outcome assessed.	Table 2-4<br>Supplementary Tables 7-8
**DISCUSSION**			
Discussion	23a	Provide a general interpretation of the results in the context of other evidence.	Page 8-10 "Summary of main results and limitations of the evidence"
	23b	Discuss any limitations of the evidence included in the review.	Page 8-11 "Summary of main results and limitations of the evidence"
	23c	Discuss any limitations of the review processes used.	Page 8-11 "Summary of main results and limitations of the

Section and Topic	Item #	Checklist item	Location where item is reported
			evidence"
	23d	Discuss implications of the results for practice, policy, and future research.	Page 11 "Implications for research"
**OTHER INFORMATION**			
Registration and protocol	24a	Provide registration information for the review, including register name and registration number, or state that the review was not registered.	Page 3<br>First paragraph in methods
	24b	Indicate where the review protocol can be accessed, or state that a protocol was not prepared.	Page 3<br>First paragraph in methods
	24c	Describe and explain any amendments to information provided at registration or in the protocol.	N.A
Support	25	Describe sources of financial or non-financial support for the review, and the role of the funders or sponsors in the review.	Page 13<br>"Funding"
Competing interests	26	Declare any competing interests of review authors.	Page 12 "Conflict of interest closure"
Availability of data, code and other materials	27	Report which of the following are publicly available and where they can be found: template data collection forms; data extracted from included studies; data used for all analyses; analytic code; any other materials used in the review.	RevMan and Covidence<br>Page 13 "Data availability statement"

From: Page MJ, McKenzie JE, Bossuyt PM, Boutron I, Hoffmann TC, Mulrow CD, et al. The PRISMA 2020 statement: an updated guideline for reporting systematic reviews. BMJ 2021;372:n71. doi: 10.1136/bmj.n71. This work is licensed under CC BY 4.0. To view a copy of this license, visit https://creativecommons.org/licenses/by/4.0/

Supplementary Table 2. Documentation Search strategies (Malmö University)

Karin Borgström, Malmö universitet
Ulrika Klintberg, Malmö universitetsbibliotek

Database and search platform	Search date	Number of records retrieved
Medline (EBSCO)	24/05/14	2840
Cinahl (EBSCO)	24/05/14	1181
Scopus (Elsevier)	24/05/14	5782

Total number of records retrieved	Duplicates	Total records to be screened
9803	3392	6411

Search strategy

A literature search was performed in the following databases: Medline (EBSCO), Cinahl (EBSCO) and Scopus (Elsevier). The last search was conducted 20240514. Deduplication was performed in Covidence. Full search strategies for all databases are available in the appendix.

Field codes

AB = Abstract; MH = Exact subject heading; MH+ = Exact subject heading, also searches subject headings below the selected one; TI = Title; TITLE-ABS-KEY = searches title, abstract and keywords; "*" = Truncation, searches word variations

Medline (EBSCO)	
**Search terms**	**Items found**
**Population**	

1. (MH "Diabetes Mellitus+")	525,089
2. TI diabetes OR AB diabetes	670,424
3. (MH "Diabetic Foot")	11,999
4. TI Diabetic OR AB Diabetic	301,579
**Population**	
5. (MH "Foot")	30,969
6. TI foot OR AB foot	139,734
**Intervention**	
7. (MH "Dermatologic Agents")	25,685
8. (MH "Keratolytic Agents")	2,804
9. (MH "Emollients")	2,365
10. (MH "Antipruritics")	1,117
11. (MH "Ointments")	13,424
12. (MH "Ointment Bases")	639
13. (MH "Liniments")	129
14. (MH "Skin Cream")	1,394
15. (MH "Gels+")	71,254
16. (MH "Hydrogels")	31,232
17. (MH "Administration, Topical")	40,734
18. (MH "Administration, Cutaneous")	25,019
19. (MH "Emulsions")	22,678
20. (MH "Ceramides+")	15,299
21. (MH "Hygroscopic Agents")	143
22. (MH "Glycerol")	27,303
23. (MH "Urea+")	126,885
24. (MH "Lactic Acid")	47,556
25. (MH "Gelatin")	15,570
26. (MH "Petrolatum+")	3,241
27. (MH "Plant Oils+")	43,452
28. (MH "Dimethylpolysiloxanes+")	6,539
29. (MH "Propylene Glycols+")	35,383
30. (MH "Lanolin")	446
31. (MH "Paraffin")	4,019
32. (MH "Cholesterol")	129,347
33. (MH "Collagen")	101,965
34. (MH "Keratins")	22,331
35. (MH "Elastin")	8,433
36. TI keratolytic agents OR AB keratolytic agents	138
37. TI emollients OR AB emollients	2,203
38. TI antipruritics OR AB antipruritics	924
39. TI ointments OR AB ointments	13,540
40. TI liniments OR AB liniments	173
41. TI gels OR AB gels	341,059
42. TI hydrogel OR AB hydrogel	62,773

43. TI cream OR AB cream	21,956
44. TI (topical OR cutaneous) OR AB (topical OR cutaneous)	290,463
45. TI lotion OR AB lotion	2,738
46. TI moisturi* OR AB moisturi*	3,658
47. TI hydrat* OR AB hydrat*	75,754
48. TI hydrogel OR AB hydrogel	62,773
49. TI salves OR AB salves	360
50. TI pastes OR AB pastes	15,728
51. TI emulsions OR AB emulsions	43,031
52. TI ceramides OR AB ceramides	16,843
53. TI hygroscopic agents OR AB hygroscopic agents	15
54. TI humectants OR AB humectants	425
55. TI glycerol OR AB glycerol	49,977
56. TI glycerin OR AB glycerin	2,845
57. TI urea OR AB urea	99,092
58. TI lactic acid OR AB lactic acid	49,650
59. TI ammonium lactate OR AB ammonium lactate	270
60. TI gelatin OR AB gelatin	32,825
61. TI hyaluronic acid OR AB hyaluronic acid	25,832
62. TI petrolatum OR AB petrolatum	1,340
63. ((TI plant OR vegetable OR castor OR coconut OR corn OR cottonseed OR linseed OR olive OR palm OR peanut OR rapeseed OR rice bran OR safflower OR sesame OR soybean) OR (AB plant OR vegetable OR castor OR coconut OR corn OR cottonseed OR linseed OR olive OR palm OR peanut OR rapeseed OR rice bran OR safflower OR sesame OR soybean)) AND (TI oil OR AB oil)	73,146
64. TI Dimethylpolysiloxanes OR AB Dimethylpolysiloxanes	149
65. TI dimethicone OR AB dimethicone	184
66. TI simethicone OR AB simethicone	333
67. TI propylene glycols OR AB propylene glycols	5,955
68. TI occlusives OR AB occlusives	31,321
69. TI lanolin OR AB lanolin	533
70. TI paraffin OR AB paraffin	59,514
71. TI cholesterol OR AB cholesterol	281,407
72. TI stearyl alcohol OR AB stearyl alcohol	150
73. TI collagen OR AB collagen	225,341
74. TI keratins OR AB keratins	18,954
75. TI elastin OR AB elastin	14,138
76. TI humidif* OR AB humidif*	5,321
77. TI foam OR AB foam	30,499
78. S1 OR S2 OR S3 OR S4	867,766
79. S5 OR S6	150,894
80. S7 OR S8 OR S9 OR S10 OR S11 OR S12 OR S13 OR S14 OR S15 OR S16 OR S17 OR S18 OR S19 OR S20 OR S21 OR S22 OR S23 OR S24 OR S25 OR S26 OR S27 OR S28 OR S29 OR S30 OR S31 OR S32 OR S33 OR S34 OR S35 OR S36 OR S37 OR S38 OR S39 OR S40 OR S41 OR S42 OR S43 OR S44 OR S45	2,035,523

OR S46 OR S47 OR S48 OR S49 OR S50 OR S51 OR S52 OR S53 OR S54 OR S55 OR S56 OR S57 OR S58 OR S59 OR S60 OR S61 OR S62 OR S63 OR S64 OR S65 OR S66 OR S67 OR S68 OR S69 OR S70 OR S71 OR S72 OR S73 OR S74 OR S75 OR S76 OR S77	
81. S78 AND S79 AND S80	2,840

Cinahl (EBSCO)

**Search terms**	**Items found**
**Population**	
1. (MH "Diabetes Mellitus+")	190,353
2. TI diabetes OR AB diabetes	210,029
3. (MH "Diabetic Foot")	10,703
4. TI Diabetic OR AB Diabetic	63,469
**Population**	
5. (MH "Foot")	13,123
6. TI foot OR AB foot	46,933
**Intervention**	
7. (MH "Dermatologic Agents")	2,467
8. (MH "Keratolytic Agents")	1,445
9. (MH "Emollients+")	2,120
10. (MH "Antipruritics+")	1,445
11. (MH "Ointments")	1,957
12. (MH "Liniments")	20
13. (MH "Creams")	2,616
14. (MH "Gels")	4,986
15. (MH "Emulsions")	809
16. (MH "Ceramides")	177
17. (MH "Propylene Glycols+")	887
18. (MH "Glycerin")	1,074
19. (MH "Urea+")	7,120
20. (MH "Lactic Acid")	3,434
21. (MH "Plant Oils+")	13,115
22. (MH "Dimethylpolysiloxanes+")	225
23. (MH "Paraffin")	278
24. (MH "Cholesterol")	18,029
25. (MH "Collagen")	12,064
26. (MH "Administration, Topical")	9,662
27. TI dermatolog* OR AB dermatolog*	15,009
28. TI keratolytic agents OR AB keratolytic agents	24
29. TI emollients OR AB emollients	632
30. TI antipruritics OR AB antipruritics	126
31. TI ointments OR AB ointments	1,880
32. TI liniments OR AB liniments	22
33. TI gels OR AB gels	12,970
34. TI hydrogel OR AB hydrogel	2,333

35. TI cream OR AB cream	4,191
36. (TI (topical OR cutaneous) OR AB (topical OR cutaneous))	41,553
37. TI lotion OR AB lotion	517
38. TI moisturi* OR AB moisturi*	822
39. TI hydrat* OR AB hydrat*	6,660
40. TI humidif* OR AB humidif*	1,260
41. TI foam OR AB foam	3,994
42. TI hydrogel OR AB hydrogel	2,333
43. TI salves OR AB salves	81
44. TI pastes OR AB pastes	1,512
45. TI emulsions OR AB emulsions	2,413
46. TI ceramides OR AB ceramides	924
47. TI hygroscopic agents OR AB hygroscopic agents	1
48. TI humectants OR AB humectants	33
49. TI glycerol OR AB glycerol	1,557
50. TI glycerin OR AB glycerin	237
51. TI urea OR AB urea	6,660
52. TI lactic acid OR AB lactic acid	2,115
53. TI ammonium lactate OR AB ammonium lactate	33
54. TI gelatin OR AB gelatin	1,557
55. TI hyaluronic acid OR AB hyaluronic acid	2,697
56. TI petrolatum OR AB petrolatum	136
57. ((TI plant OR vegetable OR castor OR coconut OR corn OR cottonseed OR linseed OR olive OR palm OR peanut OR rapeseed OR rice bran OR safflower OR sesame OR soybean) OR (AB plant OR vegetable OR castor OR coconut OR corn OR cottonseed OR linseed OR olive OR palm OR peanut OR rapeseed OR rice bran OR safflower OR sesame OR soybean)) AND (TI oil OR AB oil)	8,011
58. TI Dimethylpolysiloxanes OR AB Dimethylpolysiloxanes	5
59. TI dimethicone OR AB dimethicone	35
60. TI simethicone OR AB simethicone	101
61. TI propylene glycols OR AB propylene glycols	321
62. TI occlusives OR AB occlusives	4,070
63. TI lanolin OR AB lanolin	69
64. TI paraffin OR AB paraffin	4,671
65. TI cholesterol OR AB cholesterol	45,189
66. TI stearyl alcohol OR AB stearyl alcohol	2
67. TI collagen OR AB collagen	19,523
68. TI keratins OR AB keratins	755
69. TI elastin OR AB elastin	860
70. S1 OR S2 OR S3 OR S4	279,711
71. S5 OR S6	51,305
72. S7 OR S8 OR S9 OR S10 OR S11 OR S12 OR S13 OR S14 OR S15 OR S16 OR S17 OR S18 OR S19 OR S20 OR S21 OR S22 OR S23 OR S24 OR S25 OR S26 OR S27 OR S28 OR S29 OR S31 OR S32 OR S33 OR S34 OR S35 OR S36 OR S37 OR S38 OR S39 OR S40 OR S41 OR S42 OR S43 OR S44 OR S45 OR S46 OR S47 OR S48 OR S49 OR S50 OR S51 OR S52 OR S53 OR S54 OR S55 OR S56 OR S57 OR S58 OR S59 OR S60 OR S61 OR S62 OR S63 OR S64 OR S65 OR S66 OR S67 OR S68 OR S69 OR S70 OR S71 OR S72 OR S73 OR S74 OR S75 OR S76 OR S77 OR S78 OR S79 OR S80	217,804

73. S81 AND S82 AND S83	1181

Scopus (Elsevier)

**Search terms**	**Items found**
**Population**	
1. TITLE-ABS-KEY (diabetes)	1,192,581
2. TITLE-ABS-KEY (diabetic)	485,081
**Population**	
3. TITLE-ABS-KEY (foot)	324,828
**Intervention**	
4. TITLE-ABS-KEY (dermatolog*)	187,971
5. TITLE-ABS-KEY (keratolytic agents)	3,679
6. TITLE-ABS-KEY (emollients)	8,998
7. TITLE-ABS-KEY (antipruritics)	1,001
8. TITLE-ABS-KEY (ointments)	31,351
9. TITLE-ABS-KEY (liniments)	406
10. TITLE-ABS-KEY (cream)	52,513
11. TITLE-ABS-KEY (gels)	1,007,310
12. TITLE-ABS-KEY (hydrogels)	138,766
13. TITLE-ABS-KEY (emulsions)	169,834
14. TITLE-ABS-KEY (ceramides)	25,852
15. TITLE-ABS-KEY (hygroscopic agents)	944
16. TITLE-ABS-KEY (glycerol)	145,726
17. TITLE-ABS-KEY (urea)	288,582
18. TITLE-ABS-KEY (lactic acid)	213,222
19. TITLE-ABS-KEY (gelatin)	73,104
20. TITLE-ABS-KEY (hyaluronic acid)	64,716
21. TITLE-ABS-KEY (petrolatum)	6,488
22. TITLE-ABS-KEY (dimethylpolysiloxanes)	5,146
23. TITLE-ABS-KEY (propylene glycols)	26,676
24. TITLE-ABS-KEY (lanolin)	2,971
25. TITLE-ABS-KEY (paraffin)	136,772
26. TITLE-ABS-KEY (cholesterol)	530,051
27. TITLE-ABS-KEY (collagen)	362,003
28. TITLE-ABS-KEY (keratins)	47,339
29. TITLE-ABS-KEY (elastin)	22,895
30. TITLE-ABS-KEY (topical OR cutaneous)	508,313
31. TITLE-ABS-KEY (lotion)	5,965
32. TITLE-ABS-KEY (moisturi*)	6,747
33. TITLE-ABS-KEY (hydrat*)	288,825
34. TITLE-ABS-KEY (humidif*)	20,062

35. TITLE-ABS-KEY (foam)	148,571
36. TITLE-ABS-KEY (salves)	966
37. TITLE-ABS-KEY (pastes)	87,990
38. TITLE-ABS-KEY (humectants)	1,155
39. TITLE-ABS-KEY (glycerin)	10,356
40. TITLE-ABS-KEY (ammonium lactate)	2,288
41. (TITLE-ABS-KEY (plant OR vegetable OR castor OR coconut OR corn OR cottonseed OR linseed OR olive OR palm OR peanut OR rapeseed OR rice AND bran OR safflower OR sesame OR soybean) AND TITLE-ABS-KEY (oil))	25,379
42. TITLE-ABS-KEY (dimethicone)	409
43. TITLE-ABS-KEY (simethicone)	1,570
44. TITLE-ABS-KEY (occlusives)	129
45. TITLE-ABS-KEY (stearyl alcohol)	711
46. (TITLE-ABS-KEY (diabetes)) OR (TITLE-ABS-KEY (diabetic))	1,312,069
47. ((TITLE-ABS-KEY (dermatolog*)) OR (TITLE-ABS-KEY (keratolytic AND agents)) OR (TITLE-ABS-KEY (emollients)) OR (TITLE-ABS-KEY (antipruritics)) OR (TITLE-ABS-KEY (ointments)) OR (TITLE-ABS-KEY (liniments)) OR (TITLE-ABS-KEY (cream)) OR (TITLE-ABS-KEY (gels)) OR (TITLE-ABS-KEY (hydrogels)) OR (TITLE-ABS-KEY (emulsions)) OR (TITLE-ABS-KEY (ceramides)) OR (TITLE-ABS-KEY (hygroscopic AND agents)) OR (TITLE-ABS-KEY (glycerol)) OR (TITLE-ABS-KEY (urea)) OR (TITLE-ABS-KEY (lactic AND acid)) OR (TITLE-ABS-KEY (gelatin))) OR ((TITLE-ABS-KEY (hyaluronic AND acid)) OR (TITLE-ABS-KEY (petrolatum)) OR (TITLE-ABS-KEY (dimethylpolysiloxanes)) OR (TITLE-ABS-KEY (propylene AND glycols)) OR (TITLE-ABS-KEY (lanolin)) OR (TITLE-ABS-KEY (paraffin)) OR (TITLE-ABS-KEY (cholesterol)) OR (TITLE-ABS-KEY (collagen)) OR (TITLE-ABS-KEY (keratins)) OR (TITLE-ABS-KEY (elastin)) OR (TITLE-ABS-KEY (topical OR cutaneous)) OR (TITLE-ABS-KEY (lotion)) OR (TITLE-ABS-KEY (moisturi*)) OR (TITLE-ABS-KEY (hydrat*)) OR (TITLE-ABS-KEY (humidif*)) OR (TITLE-ABS-KEY (foam)) OR (TITLE-ABS-KEY (salves)) OR (TITLE-ABS-KEY (pastes)) OR (TITLE-ABS-KEY (humectants)) OR (TITLE-ABS-KEY (glycerin)) OR (TITLE-ABS-KEY (ammonium AND lactate)) OR (TITLE-ABS-KEY (dimethicone)) OR (TITLE-ABS-KEY (simethicone)) OR (TITLE-ABS-KEY (occlusives)) OR (TITLE-ABS-KEY (stearyl AND alcohol)))	4,080,927
48. S80 AND S81 AND S82	5782

Supplementary Table 3. Documentation Search strategies (Lund University)

Maria Björklund, Librarian / Information specialist
Library & ICT, Faculty of Medicine
Lund University
maria.bjorklund@med.lu.se

LUND UNIVERSITY
Faculty of Medicine

Search report

Search strategies

The search strategy documentation is to be cited as below, for protocol or publication in journal as appendix. The documentation is structured according to PRISMA 2020, international standard for systematic reviews. Please note that the information specialist delivers the parts required for search strategy documentation, additional method information may also be required.

See PRISMA 2020 for further information:
http://prisma-statement.org/

http://prisma-statement.org/Extensions/Searching

Databases

CENTRAL via Cochrane Library Online (Wiley, Issue 1 of 12, January 2025)

Date of search: January 29, 2025

#1 MeSH descriptor: [Diabetes Mellitus] explode all trees
47384

#2 (diabetes OR diabetic):ti,ab,kw
126767

#3 MeSH descriptor: [Foot] explode all trees
1876

#4 (foot):ti,ab,kw
22617

#5 MeSH descriptor: [Diabetic Foot] explode all trees
1612

#6 MeSH descriptor: [Dermatologic Agents] explode all trees
4978

#7 MeSH descriptor: [Keratolytic Agents] explode all trees
422

#8 MeSH descriptor: [Emollients] explode all trees
691

#9 MeSH descriptor: [Antipruritics] explode all trees
176

#10 MeSH descriptor: [Ointments] explode all trees
2326

#11 MeSH descriptor: [Ointment Bases] explode all trees
51

#12 MeSH descriptor: [Liniments] explode all trees
9

#13 MeSH descriptor: [Skin Cream] explode all trees
477

#14 MeSH descriptor: [Gels] explode all trees
3407

#15 MeSH descriptor: [Hydrogels] explode all trees
477

#16 MeSH descriptor: [Administration, Topical] explode all trees
19321

#17 MeSH descriptor: [Administration, Cutaneous] explode all trees

4714

#18 MeSH descriptor: [Emulsions] explode all trees
1267

#19 MeSH descriptor: [Ceramides] explode all trees
146

#20 MeSH descriptor: [Hygroscopic Agents] explode all trees
6

#21 MeSH descriptor: [Glycerol] explode all trees
976

#22 MeSH descriptor: [Urea] explode all trees

#23 MeSH descriptor: [Lactic Acid] explode all trees
3004

#24 MeSH descriptor: [Gelatin] explode all trees
351

#25 MeSH descriptor: [Petrolatum] explode all trees
338

#26 MeSH descriptor: [Plant Oils] explode all trees
2953

#27 MeSH descriptor: [Dimethylpolysiloxanes] explode all trees
348

#28 MeSH descriptor: [Propylene Glycols] explode all trees
1168

#29 MeSH descriptor: [Lanolin] explode all trees
40

#30 MeSH descriptor: [Paraffin] explode all trees

97

#31 MeSH descriptor: [Cholesterol] explode all trees
13218

#32 MeSH descriptor: [Collagen] explode all trees
3322

#33 MeSH descriptor: [Keratins] explode all trees
278

#34 MeSH descriptor: [Elastin] explode all trees
52

#35 (keratolytic agents OR emollients OR antipruritics OR ointment* OR liniments OR gels OR hydrogel OR cram OR topical OR cutaneous OR lotion OR moisturi* OR hydrat* OR salves OR pastes OR emulsions OR ceramides OR hygroscopic agents OR humectants OR glycerol OR glycerin OR urea OR carbamide OR lactic acid OR ammonium lactate OR gelatin OR petrolatum):ti,ab,kw

87482

#36 ((plant OR vegetable OR castor OR coconut OR corn OR cottonseed OR linseed OR olive OR palm OR peanut OR rapeseed OR rice bran OR safflower OR sesame OR soybean) AND oil):ti,ab,kw

7318

#37 (Dimethylpolysiloxanes OR dimethicone OR siimethicone OR propylene glycols OR occlusives OR lanolin OR paraffin OR cholesterol OR stearyl alcohol OR collagen OR keratins OR elastin OR humidif* OR foam):ti,ab,kw

64150

#38 ((#1 OR #2) AND (#3 OR #4)) OR #5

5526

#39 #6 OR #7 OR #8 OR #9 OR #10 OR #11 OR #12 OR #13 OR #14 OR #15 OR #16 OR #17 OR #18 OR #19 OR #20 OR #21 OR #22 OR #23 OR #24 OR #25 OR #6 OR #27 OR #28 OR #29 OR #30 OR #31 OR #32 OR #33 OR #34 OR #35 OR #36 OR #37

165417

#40 #38 AND #40
1177 of which 1151 are trial records (exported to EndNote/Covidence)

Date added to CENTRAL 19500101-20240514

1078 trials

Search strategy Embase

The search strategy documentation is to be cited as below, for protocol or publication in journal as appendix. The documentation is structured according to PRISMA 2020, international standard for systematic reviews. Please note that the information specialist delivers the parts required for search strategy documentation, additional method information may also be required.

See PRISMA 2020 for further information:

http://prisma-statement.org/

Field codes

Exp/ = subject heading (Emtree term), ab,ti= title/abstract.

“*” = Truncation, searches word variations.

Search conducted by: Maria Björklund, Librarian / Information specialist

maria.bjorklund@med.lu.se
Library & ICT, Faculty of Medicine

Lund University

Embase.com (Elsevier, 1947-present)

Date of search: January 24, 2025

#1	'diabetes mellitus'/exp	1400658
#2	diabetes:ab,ti	1101700
#3	'diabetic foot'/exp OR 'diabetic foot':ab,ti	26304
#4	diabetic:ab,ti	443683
#5	'foot'/exp	92149
#6	'foot':ab,ti	163628
#7	'dermatological agent'/exp	2116312
#8	'keratolytic agent'/exp	387751
#9	'emollient agent'/exp	8180
#10	'antipruritic agent'/exp	281032
#11	'ointment'/exp	18785
#12	'ointment base'/exp	225170
#13	'liniment'/exp	250
#14	'skin cream'/exp	1045
#15	'gel'/exp	127297
#16	'hydrogel'/exp	79525
#17	'topical drug administration'/exp	122877
#18	'cutaneous drug administration'/exp	1187
#19	'emulsion'/exp	62874
#20	'ceramide'/exp	18660
#21	'hygroscopic agent'/exp	130
#22	'glycerol'/exp	51821
#23	'urea'/exp OR 'carbamide'/exp	106522
#24	'lactic acid'/exp	114331
#25	'gelatin'/exp	36689
#26	'petrolatum'/exp	5983
#27	'plant oils'/exp	129424
#28	'dimeticone'/exp	16947
#29	'propylene glycol'/exp	12172
#30	'lanolin'/exp	1667

#31	'paraffin'/exp	50383
#32	'cholesterol'/exp	425967
#33	'collagen'/exp	287174
#34	'keratin'/exp	85094
#35	'elastin'/exp	19313
#36	'keratolytic agents':ab,ti	134
#37	emollients:ab,ti	2721
#38	antipruritics:ab,ti	102
#39	ointments:ab,ti	4758
#40	'liniment':ab,ti	183
#41	gels:ab,ti	60042
#42	hydrogel:ab,ti	61732
#43	cream:ab,ti	30266
#44	topical:ab,ti OR cutaneous:ab,ti	415703
#45	lotion:ab,ti	3441
#46	moisturi*:ab,ti	6158
#47	hydrat*:ab,ti	92790
#48	salves:ab,ti	141
#49	pastes:ab,ti	2647
#50	emulsions:ab,ti	20569
#51	ceramides:ab,ti	6927
#52	'hygroscopic agents':ab,ti	4
#53	humectants:ab,ti	346
#54	glycerol:ab,ti	62771
#55	glycerin:ab,ti	4138
#56	urea:ab,ti OR carbamide:ab,ti	134936
#57	'lactic acid':ab,ti	49641
#58	'ammonium lactate':ab,ti	130
#59	gelatin:ab,ti	41164
#60	'hyaluronic acid':ab,ti	34501
#61	petrolatum:ab,ti	2104
#62	(plant:ab,ti OR vegetable:ab,ti OR castor:ab,ti OR coconut:ab,ti OR corn:ab,ti OR cottonseed:ab,ti OR linseed:ab,ti OR olive:ab,ti OR palm:ab,ti OR peanut:ab,ti OR rapeseed:ab,ti OR 'rice bran':ab,ti OR safflower:ab,ti OR sesame:ab,ti OR soybean:ab,ti) AND oil:ab,ti	77267
#63	dimethylpolysiloxanes:ab,ti	8
#64	dimethicone:ab,ti	258
#65	simethicone:ab,ti	678
#66	'propylene glycols':ab,ti	30
#67	occlusives:ab,ti	68
#68	lanolin:ab,ti	699
#69	paraffin:ab,ti	93123

#70	cholesterol:ab,ti	397064
#71	'stearyl alcohol':ab,ti OR octadecanol:ab,ti	447
#72	collagen:ab,ti	307531
#73	keratins:ab,ti	5112
#74	elastin:ab,ti	19140
#75	humidif*:ab,ti	8563
#76	foam:ab,ti	35601
#77	#7 OR #8 OR #9 OR #10 OR #11 OR #12 OR #13 OR #14 OR #15 OR #16 OR #17 OR #18 OR #19 OR #20 OR #21 OR #22 OR #23 OR #24 OR #25 OR #26 OR #27 OR #28 OR #29 OR #30 OR #31 OR #32 OR #33 OR #34 OR #35 OR #36 OR #37 OR #38 OR #39 OR #40 OR #41 OR #42 OR #43 OR #44 OR #45 OR #46 OR #47 OR #48 OR #49 OR #50 OR #51 OR #52 OR #53 OR #54 OR #55 OR #56 OR #57 OR #58 OR #59 OR #60 OR #61 OR #62 OR #63 OR #64 OR #65 OR #66 OR #67 OR #68 OR #69 OR #70 OR #71 OR #72 OR #73 OR #74 OR #75 OR #76	4286281
#78	#1 OR #2 OR #4	1616541
#79	#5 OR #6	215638
#80	#78 AND #79	33623
#81	#3 OR #80	38797
#82	#77 AND #81	6808
#83	'conference abstract':it OR 'conference paper':it OR 'conference review':it	6136594
#84	#82 NOT #83	5397
#85	#84 AND AND [01-01-1947]/sd NOT [15-05-2024]/sd	4996

Annotation Embase:

Embase indicates approximately 1700 unique records, Covidence will identify duplicates (approximately 3600).

Main search strategy (Malmö University) was replicated using Embase specific thesaurus terms, see highlights in rows 23, 56, 71. In main search strategy, one row was redundant and therefore omitted from the Embase search (see rows 41 and 48 in main search strategy). The final combination search was altered to reduce unintended redundancy or unintended AND combinations, row 78-82. Conference abstracts and publications were filtered out.

Supplementary Table 4. Search strategies - Search Update (August 2026)

Documentation of Search Strategies – Search Update (August 2026)

Maria Björklund, Medicinska fakultetens bibliotek, Lunds universitet

Database and platform	Search dates	Number of records retrieved
Medline (EBSCO)	3 aug 2026	770
Cinahl Ultimate (EBSCO)	4 aug 2026	171
Scopus (Elsevier)	4 aug 2026	1801
Embase.com (Elsevier)	3 aug 2026	612
CENTRAL (Cochrane Library Online)	4 aug 2026	275
**Total (before deduplication)**		**3629**
**After deduplication in Covidence**		**2189**

Field codes

AB = Abstract; MH = Exact subject heading; MH+ = Exact subject heading, also searches subject headings below the selected one; TI = Title; TITLE-ABS-KEY = searches title, abstract and keywords; "*" = Truncation, searches word variations.

Medline (EBSCO) Date of search: August 3, 2026	
**Search terms**	**Items found**
**Population**	
82. (MH "Diabetes Mellitus+")	574766
83. TI diabetes OR AB diabetes	780044
84. (MH "Diabetic Foot")	14274
85. TI Diabetic OR AB Diabetic	340093
**Population**	
86. (MH "Foot")	51281
87. TI foot OR AB foot	157400
**Intervention**	
88. (MH "Dermatologic Agents")	27001
89. (MH "Keratolytic Agents")	2,8043
90. (MH "Emollients")	2494
91. (MH "Antipruritics")	1182
92. (MH "Ointments")	13671
93. (MH "Ointment Bases")	641
94. (MH "Liniments")	129
95. (MH "Skin Cream")	1750
96. (MH "Gels+")	88640
97. (MH "Hydrogels")	44246
98. (MH "Administration, Topical")	42526
99. (MH "Administration, Cutaneous")	27181
100. (MH "Emulsions")	26490
101. (MH "Ceramides+")	16061
102. (MH "Hygroscopic Agents")	146
103. (MH "Glycerol")	28329
104. (MH "Urea+")	133198
105. (MH "Lactic Acid")	51258
106. (MH "Gelatin")	18399
107. (MH "Petrolatum+")	3329
108. (MH "Plant Oils+")	48033
109. (MH "Dimethylpolysiloxanes+")	7187
110. (MH "Propylene Glycols+")	36373
111. (MH "Lanolin")	454
112. (MH "Paraffin")	4158

113.	(MH "Cholesterol")	133352
114.	(MH "Collagen")	106265
115.	(MH "Keratins")	22798
116.	(MH "Elastin")	8882
117.	TI keratolytic agents OR AB keratolytic agents	138
118.	TI emollients OR AB emollients	2524
119.	TI antipruritics OR AB antipruritics	1068
120.	TI ointments OR AB ointments	14746
121.	TI liniments OR AB liniments	190
122.	TI gels OR AB gels	364388
123.	TI hydrogel OR AB hydrogel	87672
124.	TI cream OR AB cream	24571
125.	TI (topical OR cutaneous) OR AB (topical OR cutaneous)	323410
126.	TI lotion OR AB lotion	3003
127.	TI moisturi* OR AB moisturi*	4410
128.	TI hydrat* OR AB hydrat*	87631
129.	TI salves OR AB salves	404
130.	TI pastes OR AB pastes	17957
131.	TI emulsions OR AB emulsions	50399
132.	TI ceramides OR AB ceramides	18867
133.	TI hygroscopic agents OR AB hygroscopic agents	139
134.	TI humectants OR AB humectants	494
135.	TI glycerol OR AB glycerol	54445
136.	TI glycerin OR AB glycerin	3201
137.	TI urea OR AB urea	110619
138.	TI lactic acid OR AB lactic acid	60231
139.	TI ammonium lactate OR AB ammonium lactate	725
140.	TI gelatin OR AB gelatin	38738
141.	TI hyaluronic acid OR AB hyaluronic acid	32592
142.	TI petrolatum OR AB petrolatum	1422
143.	((TI plant OR vegetable OR castor OR coconut OR corn OR cottonseed OR linseed OR olive OR palm OR peanut OR rapeseed OR rice bran OR safflower OR sesame OR soybean) OR (AB plant OR vegetable OR castor OR coconut OR corn OR cottonseed OR linseed OR olive OR palm OR peanut OR rapeseed OR rice bran OR safflower OR sesame OR soybean)) AND (TI oil OR AB oil)	84070
144.	TI Dimethylpolysiloxanes OR AB Dimethylpolysiloxanes	160
145.	TI dimethicone OR AB dimethicone	207
146.	TI simethicone OR AB simethicone	380
147.	TI propylene glycols OR AB propylene glycols	6800
148.	TI occlusives OR AB occlusives	33928
149.	TI lanolin OR AB lanolin	573
150.	TI paraffin OR AB paraffin	63844
151.	TI cholesterol OR AB cholesterol	311887
152.	TI stearyl alcohol OR AB stearyl alcohol	180

153.	TI collagen OR AB collagen	251877
154.	TI keratins OR AB keratins	20641
155.	TI elastin OR AB elastin	15486
156.	TI humidif* OR AB humidif*	5850
157.	TI foam OR AB foam	36414
158.	S1 OR S2 OR S3 OR S4	994126
159.	S5 OR S6	183399
160.	S7 OR S8 OR S9 OR S10 OR S11 OR S12 OR S13 OR S14 OR S15 OR S16 OR S17 OR S18 OR S19 OR S20 OR S21 OR S22 OR S23 OR S24 OR S25 OR S26 OR S27 OR S28 OR S29 OR S30 OR S31 OR S32 OR S33 OR S34 OR S35 OR S36 OR S37 OR S38 OR S39 OR S40 OR S41 OR S42 OR S43 OR S44 OR S45 OR S46 OR S47 OR S48 OR S49 OR S50 OR S51 OR S52 OR S53 OR S54 OR S55 OR S56 OR S57 OR S58 OR S59 OR S60 OR S61 OR S62 OR S63 OR S64 OR S65 OR S66 OR S67 OR S68 OR S69 OR S70 OR S71 OR S72 OR S73 OR S74 OR S75 OR S76	2250556
161.	S77 AND S78 AND S79	3624
162.	S80 AND Publication date filter 05/2024-/12/2026	**770**

Annotation Medline: in previous search strategy row 42 was accidentally duplicated in row 48, hence previous row 48 was deleted in the updated search and the numbering of search rows was updated accordingly.

Cinahl Ultimate (EBSCO), Date of search: August 4, 0226	
**Search terms**	**Items found**
**Population**	
74. (MH "Diabetes Mellitus+")	207157
75. TI diabetes OR AB diabetes	233656
76. (MH "Diabetic Foot")	12121
77. TI Diabetic OR AB Diabetic	69557
**Population**	
78. (MH "Foot")	14521
79. TI foot OR AB foot	52576
**Intervention**	
80. (MH "Dermatologic Agents")	2653
81. (MH "Keratolytic Agents")	336
82. (MH "Emollients+")	2290
83. (MH "Antipruritics+")	1635
84. (MH "Ointments")	2184
85. (MH "Liniments")	22
86. (MH "Creams")	2862
87. (MH "Gels")	5517
88. (MH "Emulsions")	872
89. (MH "Ceramides")	237
90. (MH "Propylene Glycols+")	989
91. (MH "Glycerin")	1174
92. (MH "Urea+")	7563
93. (MH "Lactic Acid")	3654
94. (MH "Plant Oils+")	14145
95. (MH "Dimethylpolysiloxanes+")	246
96. (MH "Paraffin")	316
97. (MH "Cholesterol")	19379
98. (MH "Collagen")	12724
99. (MH "Administration, Topical")	10652
100. TI dermatolog* OR AB dermatolog*	17893
101. TI keratolytic agents OR AB keratolytic agents	37
102. TI emollients OR AB emollients	688
103. TI antipruritics OR AB antipruritics	132
104. TI ointments OR AB ointments	2052
105. TI liniments OR AB liniments	25
106. TI gels OR AB gels	13941
107. TI hydrogel OR AB hydrogel	2757
108. TI cream OR AB cream	4863
109. (TI (topical OR cutaneous) OR AB (topical OR cutaneous))	46171
110. TI lotion OR AB lotion	609
111. TI moisturi* OR AB moisturi*	982
112. TI hydrat* OR AB hydrat*	7624

113.	TI humidif* OR AB humidif*	1415
114.	TI foam OR AB foam	4502
115.	TI hydrogel OR AB hydrogel	2757
116.	TI salves OR AB salves	90
117.	TI pastes OR AB pastes	1657
118.	TI emulsions OR AB emulsions	2669
119.	TI ceramides OR AB ceramides	1028
120.	TI hygroscopic agents OR AB hygroscopic agents	11
121.	TI humectants OR AB humectants	41
122.	TI glycerol OR AB glycerol	1659
123.	TI glycerin OR AB glycerin	344
124.	TI urea OR AB urea	7234
125.	TI lactic acid OR AB lactic acid	2468
126.	TI ammonium lactate OR AB ammonium lactate	24
127.	TI gelatin OR AB gelatin	1987
128.	TI hyaluronic acid OR AB hyaluronic acid	3109
129.	TI petrolatum OR AB petrolatum	146
130.	((TI plant OR vegetable OR castor OR coconut OR corn OR cottonseed OR linseed OR olive OR palm OR peanut OR rapeseed OR rice bran OR safflower OR sesame OR soybean) OR (AB plant OR vegetable OR castor OR coconut OR corn OR cottonseed OR linseed OR olive OR palm OR peanut OR rapeseed OR rice bran OR safflower OR sesame OR soybean)) AND (TI oil OR AB oil)	8757
131.	TI Dimethylpolysiloxanes OR AB Dimethylpolysiloxanes	5
132.	TI dimethicone OR AB dimethicone	43
133.	TI simethicone OR AB simethicone	119
134.	TI propylene glycols OR AB propylene glycols	345
135.	TI occlusives OR AB occlusives	4483
136.	TI lanolin OR AB lanolin	77
137.	TI paraffin OR AB paraffin	4741
138.	TI cholesterol OR AB cholesterol	48537
139.	TI stearyl alcohol OR AB stearyl alcohol	3
140.	TI collagen OR AB collagen	20776
141.	TI keratins OR AB keratins	817
142.	TI elastin OR AB elastin	908
143.	S1 OR S2 OR S3 OR S4	312120
144.	S5 OR S6	57338
145.	S7 OR S8 OR S9 OR S10 OR S11 OR S12 OR S13 OR S14 OR S15 OR S16 OR S17 OR S18 OR S19 OR S20 OR S21 OR S22 OR S23 OR S24 OR S25 OR S26 OR S27 OR S28 OR S29 OR S31 OR S32 OR S33 OR S34 OR S35 OR S36 OR S37 OR S38 OR S39 OR S40 OR S41 OR S42 OR S43 OR S44 OR S45 OR S46 OR S47 OR S48 OR S49 OR S50 OR S51 OR S52 OR S53 OR S54 OR S55 OR S56 OR S57 OR S58 OR S59 OR S60 OR S61 OR S62 OR S63 OR S64 OR S65 OR S66 OR S67 OR S68 OR S69	237199
146.	S70 AND S71 AND S72	1331
147.	S73 AND Publication date filter 05/2024-12/2026	**171**

Scopus (Elsevier), Date of search: August 4 2026	
**Search terms**	**Items found**
**Population**	
49. TITLE-ABS-KEY (diabetes)	1,192,581
50. TITLE-ABS-KEY (diabetic)	485,081
**Population**	
51. TITLE-ABS-KEY (foot)	324,828
**Intervention**	
52. TITLE-ABS-KEY (dermatolog*)	187,971
53. TITLE-ABS-KEY (keratolytic agents)	3,679
54. TITLE-ABS-KEY (emollients)	10151
55. TITLE-ABS-KEY (antipruritics)	1058
56. TITLE-ABS-KEY (ointments)	34048
57. TITLE-ABS-KEY (liniments)	451
58. TITLE-ABS-KEY (cream)	60332
59. TITLE-ABS-KEY (gels)	1113578
60. TITLE-ABS-KEY (hydrogels)	187123
61. TITLE-ABS-KEY (emulsions)	194722
62. TITLE-ABS-KEY (ceramides)	28890
63. TITLE-ABS-KEY (hygroscopic agents)	1111
64. TITLE-ABS-KEY (glycerol)	161307
65. TITLE-ABS-KEY (urea)	332602
66. TITLE-ABS-KEY (lactic acid)	248092
67. TITLE-ABS-KEY (gelatin)	85663
68. TITLE-ABS-KEY (hyaluronic acid)	77989
69. TITLE-ABS-KEY (petrolatum)	7168
70. TITLE-ABS-KEY (dimethylpolysiloxanes)	5682
71. TITLE-ABS-KEY (propylene glycols)	29251
72. TITLE-ABS-KEY (lanolin)	3159
73. TITLE-ABS-KEY (paraffin)	147573
74. TITLE-ABS-KEY (cholesterol)	603525
75. TITLE-ABS-KEY (collagen)	407577
76. TITLE-ABS-KEY (keratins)	50842
77. TITLE-ABS-KEY (elastin)	26584
78. TITLE-ABS-KEY (topical OR cutaneous)	563992
79. TITLE-ABS-KEY (lotion)	6692
80. TITLE-ABS-KEY (moisturi*)	8447
81. TITLE-ABS-KEY (hydrat*)	334434
82. TITLE-ABS-KEY (humidif*)	23360

83. TITLE-ABS-KEY (foam)	175067
84. TITLE-ABS-KEY (salves)	1095
85. TITLE-ABS-KEY (pastes)	101417
86. TITLE-ABS-KEY (humectants)	1330
87. TITLE-ABS-KEY (glycerin)	11428
88. TITLE-ABS-KEY (ammonium lactate)	2495
89. (TITLE-ABS-KEY (plant OR vegetable OR castor OR coconut OR corn OR cottonseed OR linseed OR olive OR palm OR peanut OR rapeseed OR rice AND bran OR safflower OR sesame OR soybean) AND TITLE-ABS-KEY (oil))	29548
90. TITLE-ABS-KEY (dimethicone)	455
91. TITLE-ABS-KEY (simethicone)	1749
92. TITLE-ABS-KEY (occlusives)	144
93. TITLE-ABS-KEY (stearyl alcohol)	765
94. 1 OR 2	1546689
95. 4 OR 5 OR 6 OR 7 OR 8 OR 9 OR 10 OR 11 OR 12 OR 13 OR 14 OR 15 OR 16 OR 17 OR 18 OR 19 OR 20 OR 21 OR 22 OR 23 OR 24 OR 25 OR 26 OR 27 OR 28 OR 29 OR 31 OR 32 OR 33 OR 34 OR 35 OR 36 OR 37 OR 38 OR 39 OR 40 OR 41 OR 42 OR 43 OR 44 OR 45	4637749
96. 3 AND 46 AND 47	7411
97. 48 AND Publication date filter 2024-2026	**1801**

Annotation Scopus: Date filter in Scopus only allows filter on year, not month/day

Search strategy Embase and CENTRAL

The search strategy documentation is to be cited as below, for protocol or publication in journal as appendix. The documentation is structured according to PRISMA 2020, international standard for systematic reviews. Please note that the information specialist delivers the parts required for search strategy documentation, additional method information may also be required.

See PRISMA 2020 for further information:

http://prisma-statement.org/

Field codes

Exp/ = subject heading (Emtree term), ab,ti= title/abstract.

“*” = Truncation, searches word variations.

Search conducted by: Maria Björklund, Librarian / Information specialist

maria.bjorklund@med.lu.se
Library & ICT, Faculty of Medicine

Lund University

Embase.com (Elsevier, 1947-present)

Date of updated search: August 3, 2026

#1	'diabetes mellitus'/exp	1618680
#2	diabetes:ab,ti	1270987
#3	'diabetic foot'/exp OR 'diabetic foot':ab,ti	31837
#4	diabetic:ab,ti	497528
#5	'foot'/exp	99336
#6	'foot':ab,ti	185140
#7	'dermatological agent'/exp	2355898
#8	'keratolytic agent'/exp	422612
#9	'emollient agent'/exp	9225
#10	'antipruritic agent'/exp	306187
#11	'ointment'/exp	21193
#12	'ointment base'/exp	246823
#13	'liniment'/exp	270
#14	'skin cream'/exp	1183
#15	'gel'/exp	155223

#16	'hydrogel'/exp	98688
#17	'topical drug administration'/exp	135367
#18	'cutaneous drug administration'/exp	1312
#19	'emulsion'/exp	72448
#20	'ceramide'/exp	20924
#21	'hygroscopic agent'/exp	144
#22	'glycerol'/exp	56512
#23	'urea'/exp OR 'carbamide'/exp	119127
#24	'lactic acid'/exp	133146
#25	'gelatin'/exp	41513
#26	'petrolatum'/exp	6697
#27	'plant oils'/exp	144466
#28	'dimeticone'/exp	18873
#29	'propylene glycol'/exp	13397
#30	'lanolin'/exp	1795
#31	'paraffin'/exp	53283
#32	'cholesterol'/exp	478965
#33	'collagen'/exp	315721
#34	'keratin'/exp	92302
#35	'elastin'/exp	20974
#36	'keratolytic agents':ab,ti	155
#37	emollients:ab,ti	3181
#38	antipruritics:ab,ti	112
#39	ointments:ab,ti	5219
#40	'liniment':ab,ti	202
#41	gels:ab,ti	64278
#42	hydrogel:ab,ti	78074
#43	cream:ab,ti	35293
#44	topical:ab,ti OR cutaneous:ab,ti	464134
#45	lotion:ab,ti	3989
#46	moisturi*:ab,ti	7591
#47	hydrat*:ab,ti	105695
#48	salves:ab,ti	149
#49	pastes:ab,ti	2933
#50	emulsions:ab,ti	23430
#51	ceramides:ab,ti	8023
#52	'hygroscopic agents':ab,ti	8
#53	humectants:ab,ti	346408
#54	glycerol:ab,ti	67154
#55	glycerin:ab,ti	4667
#56	urea:ab,ti OR carbamide:ab,ti	148782
#57	'lactic acid':ab,ti	56281

#58	'ammonium lactate':ab,ti	152
#59	gelatin:ab,ti	46398
#60	'hyaluronic acid':ab,ti	41393
#61	petrolatum:ab,ti	2303
#62	(plant:ab,ti OR vegetable:ab,ti OR castor:ab,ti OR coconut:ab,ti OR corn:ab,ti OR cottonseed:ab,ti OR linseed:ab,ti OR olive:ab,ti OR palm:ab,ti OR peanut:ab,ti OR rapeseed:ab,ti OR 'rice bran':ab,ti OR safflower:ab,ti OR sesame:ab,ti OR soybean:ab,ti) AND oil:ab,ti	85644
#63	dimethylpolysiloxanes:ab,ti	8
#64	dimethicone:ab,ti	294
#65	simethicone:ab,ti	856
#66	'propylene glycols':ab,ti	31
#67	occlusives:ab,ti	78
#68	lanolin:ab,ti	775
#69	paraffin:ab,ti	100093
#70	cholesterol:ab,ti	436405
#71	'stearyl alcohol':ab,ti OR octadecanol:ab,ti	482
#72	collagen:ab,ti	337494
#73	keratins:ab,ti	5366
#74	elastin:ab,ti	20728
#75	humidif*:ab,ti	9734
#76	foam:ab,ti	40680
#77	#7 OR #8 OR #9 OR #10 OR #11 OR #12 OR #13 OR #14 OR #15 OR #16 OR #17 OR #18 OR #19 OR #20 OR #21 OR #22 OR #23 OR #24 OR #25 OR #26 OR #27 OR #28 OR #29 OR #30 OR #31 OR #32 OR #33 OR #34 OR #35 OR #36 OR #37 OR #38 OR #39 OR #40 OR #41 OR #42 OR #43 OR #44 OR #45 OR #46 OR #47 OR #48 OR #49 OR #50 OR #51 OR #52 OR #53 OR #54 OR #55 OR #56 OR #57 OR #58 OR #59 OR #60 OR #61 OR #62 OR #63 OR #64 OR #65 OR #66 OR #67 OR #68 OR #69 OR #70 OR #71 OR #72 OR #73 OR #74 OR #75 OR #76	4757770
#78	#1 OR #2 OR #4	1862314
#79	#5 OR #6	240681
#80	#78 AND #79	40288
#81	#3 OR #80	46124
#82	#77 AND #81	8630
#83	'conference abstract':it OR 'conference paper':it OR 'conference review':it	61917900
#84	#82 NOT #83	7016
#85	#84 AND AND [01-05-2024]/sd NOT [01-01-2027]/sd	2072
#86	#85 #87 AND [embase]/lim NOT ([embase]/lim AND [medline]/lim)	**612**

Annotation Embase:

Conference abstracts and publications were filtered out.

CENTRAL via Cochrane Library Online (Wiley, Issue 8 of 12, August 2026)

Date of search: August 4, 2026

#1	MeSH descriptor: [Diabetes Mellitus] explode all trees	49476
#2	(diabetes OR diabetic):ti,ab,kw	138019
#3	MeSH descriptor: [Foot] explode all trees	1978
#4	(foot):ti,ab,kw	25767
#5	MeSH descriptor: [Diabetic Foot] explode all trees	1752
#6	MeSH descriptor: [Dermatologic Agents] explode all trees	4974
#7	MeSH descriptor: [Keratolytic Agents] explode all trees	412
#8	MeSH descriptor: [Emollients] explode all trees	666
#9	MeSH descriptor: [Antipruritics] explode all trees	174
#10	MeSH descriptor: [Ointments] explode all trees	2260
#11	MeSH descriptor: [Ointment Bases] explode all trees	52
#12	MeSH descriptor: [Liniments] explode all trees	9
#13	MeSH descriptor: [Skin Cream] explode all trees	551
#14	MeSH descriptor: [Gels] explode all trees	3438
#15	MeSH descriptor: [Hydrogels] explode all trees	498
#16	MeSH descriptor: [Administration, Topical] explode all trees	19007
#17	MeSH descriptor: [Administration, Cutaneous] explode all trees	4659
#18	MeSH descriptor: [Emulsions] explode all trees	1235
#19	MeSH descriptor: [Ceramides] explode all trees	154
#20	MeSH descriptor: [Hygroscopic Agents] explode all trees	6
#21	MeSH descriptor: [Glycerol] explode all trees	965
#22	MeSH descriptor: [Urea] explode all trees	6669
#23	MeSH descriptor: [Lactic Acid] explode all trees	3114
#24	MeSH descriptor: [Gelatin] explode all trees	343
#25	MeSH descriptor: [Petrolatum] explode all trees	340
#26	MeSH descriptor: [Plant Oils] explode all trees	2947
#27	MeSH descriptor: [Dimethylpolysiloxanes] explode all trees	344
#28	MeSH descriptor: [Propylene Glycols] explode all trees	1132

#29 MeSH descriptor: [Lanolin] explode all trees39

#30 MeSH descriptor: [Paraffin] explode all trees 96

#31 MeSH descriptor: [Cholesterol] explode all trees 12932

#32 MeSH descriptor: [Collagen] explode all trees 3403

#33 MeSH descriptor: [Keratins] explode all trees 286

#34 MeSH descriptor: [Elastin] explode all trees 56

#35 (keratolytic agents OR emollients OR antipruritics OR ointment* OR liniments OR gels OR hydrogel OR cram OR topical OR cutaneous OR lotion OR moisturi* OR hydrat* OR salves OR pastes OR emulsions OR ceramides OR hygroscopic agents OR humectants OR glycerol OR glycerin OR urea OR carbamide OR lactic acid OR ammonium lactate OR gelatin OR petrolatum):ti,ab,kw

93173

#36 ((plant OR vegetable OR castor OR coconut OR corn OR cottonseed OR linseed OR olive OR palm OR peanut OR rapeseed OR rice bran OR safflower OR sesame OR soybean) AND oil):ti,ab,kw

7663

#37 (Dimethylpolysiloxanes OR dimethicone OR siimethicone OR propylene glycols OR occlusives OR lanolin OR paraffin OR cholesterol OR stearyl alcohol OR collagen OR keratins OR elastin OR humidif* OR foam):ti,ab,kw

67667

#38 ((#1 OR #2) AND (#3 OR #4)) OR #5 6281

#39 #6 OR #7 OR #8 OR #9 OR #10 OR #11 OR #12 OR #13 OR #14 OR #15 OR #16
25970

#40 #17 OR #18 OR #19 OR #20 OR #21 OR #22 OR #23 OR #24 OR #25 OR #26
19778

#41 #27 OR #28 OR #29 OR #30 OR #31 OR #32 OR #33 OR #34 OR #35 OR #36 OR #37
161966

#42 #39 OR #40 OR #41 174533

#43 #38 AND #42

1343

1343 of which 1317 are trial records

Date added to CENTRAL 2024-05-01 to2026-12-31

275 trials

Supplementary Table 5. Details for all trials regarding intervention and control formulations

Characteristics	Baker	Carter	Federici 2015	Federici 2012	Garrigue	Gin	Glonek	Martini	Pham	Quattresooz RCT A	Quattresooz RCT B	Schultze-Walter
**Intervention formulation**	**10% urea**, Allpresan 3(Neubourg Skin Care, Greven) n= 26	**25% urea** Scholl Cracked Heel Repair Cream K+ n= 24	Ureadin (**urea 5%,** arginine 0.5% and carnosine 0.01%) n= 25	Ureadin (**urea 5%,** arginine 0.5% and carnosine 0.01%) n=20	Pedimed - 10% glycerine, **5% urea**, lactamide (moisturizing agents), and 8% petrolatum and dimethiconol (occlusive agents) in an emulsion base n=54	Pedimed - 10% glycerine, **5% urea**, lactamide (moisturizing agents), and 8% petrolatum and dimethiconol (occlusive agents) in an emulsion base n=80	Plant-based anionic polar phospholipid (APP) Blend of the natural anionic phosphatides: phos-phatidylglycerol, cardiolipin, phosphatidic acid, phosphatidylserine, and phosphatidylinositol. n=49	Dexeryl - glycerol15%, liquid and soft paraffin 10%, glycerol monostearate, stearic acid, polydimethylcyclosiloxane, silicone oil, macrogol 600, trolamine, propyl parahydroxybenzoate and purified water. n=54	Atrac-Tain® cream, (Coloplast Corporation, Marietta, Ga.) **10% urea** and 4% lactic acid in a nongreasy emulsion base. n=30	Non-greasy base enriched with 1.5% chitin-glucan (KiOsmetine CG, KitoZyme, Herstal, Belgium). n=30	Gl ycerol-enriched commercially available moisturizers, namely the 'crème pieds' Formule norvégienne(Neutrogena, France) n=30	Allpresan diabetic Original Schaum-Creme INTENSIV: aqua, **urea**, butane, decyl oleate, otyldodecanol, cetearyl alcohol, propane, stearic acid, propylene glycol, glycerin, glyceryl stearate, panthenol, saccharide isomerate, undecyl alcohol, allantoin, palm glycerides, potassium

													lauroyl wheat amino, acids, capryloyl glycine, sodium lauroyl wheat amino acids, sodium citrate, citric acid. n=49
**Control formulation**	Participants regular cream. **Aqueous cream** (made to the British Pharmacopoeia formulation), **E45** (Reckitt Benckiser, Slough), **Diprobase** (Schering–Plough, Welwyn Garden City)	Aqueous cream BP 30% w/w (ingredients include: cetostearyl alcohol, sodium lauryl sulphate, liquid paraffin and white soft paraffin, phenoxyethanol 1% w/w and purified water to 100%). n= 24	Glycerol-based (40%) emollient cream (Neutrogena Norwegian Formula Dermatological Cream) n=25	Glycerol-based (15%) emollient cream containing also vaseline (8%) (Dexeryl, Pierre Fabre) n=20	Emulsion base with none of the active ingredients (otherwise identical to Pedimed) n=54	Emulsion base with none of the active ingredients (otherwise identical to Pedimed) n=87	Mineral oil hydrocarbon (petrolatum)-based (MHB) Eucerin® n=49	Vehicle-glycerol monostearate, stearic acid, polydimethylcyclosiloxane, silicone oil, macrogol 600, trolamine, propyl parahydroxybenzoate and purified water. n=54	The vehicle was the nongreasy emulsion base, similar in texture, color, and odor without the urea and lactic acid. n=30	The vehicle: similar in texture, color and odor, but without chitin-glucan. n=30	Glycerol-enriched commercially available moisturizers ‘crème hydratante’ Cetaphil, (Galderma, France). n=30	Aqua, butane, propane, cetyl alcohol, stearic acid, caprylyl glycol, palm glycerides, sodium lauroyl wheat amino acids, capryloyl glycine. n=49

	**Unguentum** Merck (Hermal, Reinbeck) n=26											
**Dose and frequency**	Not reported Twice daily	1 g (on heel) Twice daily	2.5 g Twice daily	Not reported Twice daily	1 g Twice daily	1 g Twice daily	Not reported Twice daily	Not reported Twice daily	Not reported Twice daily	Not reported Once daily	Not reported Once daily	Not reported Twice daily

Supplementary Table 6. Descriptive Characteristics of Included Studies

Characteristics	Baker	Carter	Federici 2015	Federici 2012	Garrigue	Gin	Glonek	Martini	Pham	Quattresooz RCT A & B	Schultze-Walter
Blinding	Single - Assessor blinded	No information	Single - Assessor blinded	Single - Assessor blinded	Double blind	Double blind	Double blind	Double blind	Double blind	Double blind	Double blind
Within-person study design	Yes	Yes	No	No	Yes	No	Yes	Yes	Yes	Yes	Yes
Mean age	60	68.8	Intervention: 62<br>Control: 61	Intervention: 66<br>Control: 58	57	Overall: 63,6	70.5	58.3	62	Not reported	59
Male %	46%	68%	Intervention: 32 %<br>Control: 48%	Intervention: 50<br>Control: 30	44.4 %	Overall: 49,7	35.2%	48.2%	55%	0%	33%
Diabetes duration Mean years	16	Not reported	Intervention: 12<br>Control: 11	Intervention: 14<br>Control: 9	Type I 28.7<br>Type II 13.8	Overall: 7.3	12.6	11.6	12.4	Not reported	Not reported
Type of diabetes	I & II	II	II	II	I & II	I & II	I & II	I & II	I & II	I & II	I & II
Informed consent	Not reported	Yes	Yes	Yes	Yes	Yes	Yes	Yes	Yes	Yes	Yes

Supplementary Table 7. Summary of findings: Humectant (intervention) compared to Standard base (control) for foot xerosis in people with diabetes

Outcomes	Anticipated absolute effects* (95% CI)		Relative effect (95% CI)	№ of participants (studies)	Certainty of the evidence (GRADE)	Comments
	Risk with Standard base (control)	Risk with Humectant (intervention)				
Change from baseline in xerosis grade after two weeks of treatment	-	SMD **0.67 SD lower** (1.18 lower to 0.17 lower)	-	404 participants 641 feet (7 RCTs)	⊕⊕⊕◯ Moderate[a,b,c,d,e]	Humectant (intervention) probably results in a slight reduction in change from baseline in xerosis grade after two weeks of treatment.
Change from baseline in xerosis grade after four weeks of treatment	-	SMD **0.36 lower** (0.53 lower to 0.19 lower)	-	354 participants 541 feet (5 RCTs)	⊕⊕⊕⊕ High[c,d,e,f,g]	Humectant (intervention) results in a slight reduction in change from baseline in xerosis grade after four weeks of treatment.

***The risk in the intervention group** (and its 95% confidence interval) is based on the assumed risk in the comparison group and the **relative effect** of the intervention (and its 95% CI).

CI: confidence interval; **SMD:** standardised mean difference

GRADE Working Group grades of evidence
High certainty: we are very confident that the true effect lies close to that of the estimate of the effect.
Moderate certainty: we are moderately confident in the effect estimate: the true effect is likely to be close to the estimate of the effect, but there is a possibility that it is substantially different.
Low certainty: our confidence in the effect estimate is limited: the true effect may be substantially different from the estimate of the effect.
Very low certainty: we have very little confidence in the effect estimate: the true effect is likely to be substantially different from the estimate of effect.

Explanations

[a]One study (Carter et al.) had a high risk of bias but contributed little weight. All studies showed some concern for selective reporting only because no protocols were available, which is expected given their age. We found no indication of actual selective reporting, so we judged this as not a serious concern.

[b]Two studys (Baker et al. and Carter et al.) are the reasons for the inconsistency and high heterogeneity but they have low weights and point in the same direction why we judge as seroius and not very serious concerns

[c]Study design, population, and outcomes are directly applicable to the clinical question; there is no reason for downgrading

[d]The results are based on sufficiently large studies with narrow confidence intervals and clear effects.

[e]We only included RCT:s. Only observational studies are evaluated in this domain.

[f]Most studies show a low risk of bias, and any potential limitations are considered minor and not relevant to the outcomes.

[g]The results are consistent across studies with similar designs and populations.

Supplementary Table 8. Summary of findings: Urea (intervention) compared to Standard base (control) for foot xerosis in people with diabetes

Outcomes	Anticipated absolute effects* (95% CI)		Relative effect (95% CI)	№ of participants (studies)	Certainty of the evidence (GRADE)	Comments
	Risk with Standard base (control)	Risk with Urea (intervention)				
Change from baseline in xerosis grade after two weeks of treatment	-	SMD **0.77 SD lower** (1.35 lower to 0.19 lower)	-	350 participants 533 feet (6 RCTs)	⊕⊕⊕◯ Moderate[a,b,c,d,e]	Urea (intervention) probably reduces change from baseline in xerosis grade after two weeks of treatment slightly.
Change from baseline in xerosis grade after four weeks of treatment	-	SMD **0.36 lower** (0.55 lower to 0.17 lower)	-	300 participants 433 feet (4 RCTs)	⊕⊕⊕⊕ High[c,d,e,f,g]	Urea (intervention) results in a slight reduction in change from baseline in xerosis grade after four weeks of treatment.

***The risk in the intervention group** (and its 95% confidence interval) is based on the assumed risk in the comparison group and the **relative effect** of the intervention (and its 95% CI).

CI: confidence interval; **SMD:** standardised mean difference

GRADE Working Group grades of evidence
High certainty: we are very confident that the true effect lies close to that of the estimate of the effect.
Moderate certainty: we are moderately confident in the effect estimate: the true effect is likely to be close to the estimate of the effect, but there is a possibility that it is substantially different.
Low certainty: our confidence in the effect estimate is limited: the true effect may be substantially different from the estimate of the effect.
Very low certainty: we have very little confidence in the effect estimate: the true effect is likely to be substantially different from the estimate of effect.

Explanations

[a]One study (Carter et al.) had a high risk of bias but contributed little weight. All studies showed some concern for selective reporting only because no protocols were available, which is expected given their age. We found no indication of actual selective reporting, so we judged this as not a serious concern.

[b]Two studys (Baker et al. and Carter et al.) are the reasons for the inconsistency and high heterogeneity but they have low weights and point in the same direction why we judge as seroius and not very serious concerns

[c]Study design, population, and outcomes are directly applicable to the clinical question; there is no reason for downgrading

[d]The results are based on sufficiently large studies with narrow confidence intervals and clear effects.

[e]We only included RCT:s. Only observational studies are evaluated in this domain.

[f]Most studies show a low risk of bias, and any potential limitations are considered minor and not relevant to the outcomes.

[g]The results are consistent across studies with similar designs and populations.

Supplementary Figures

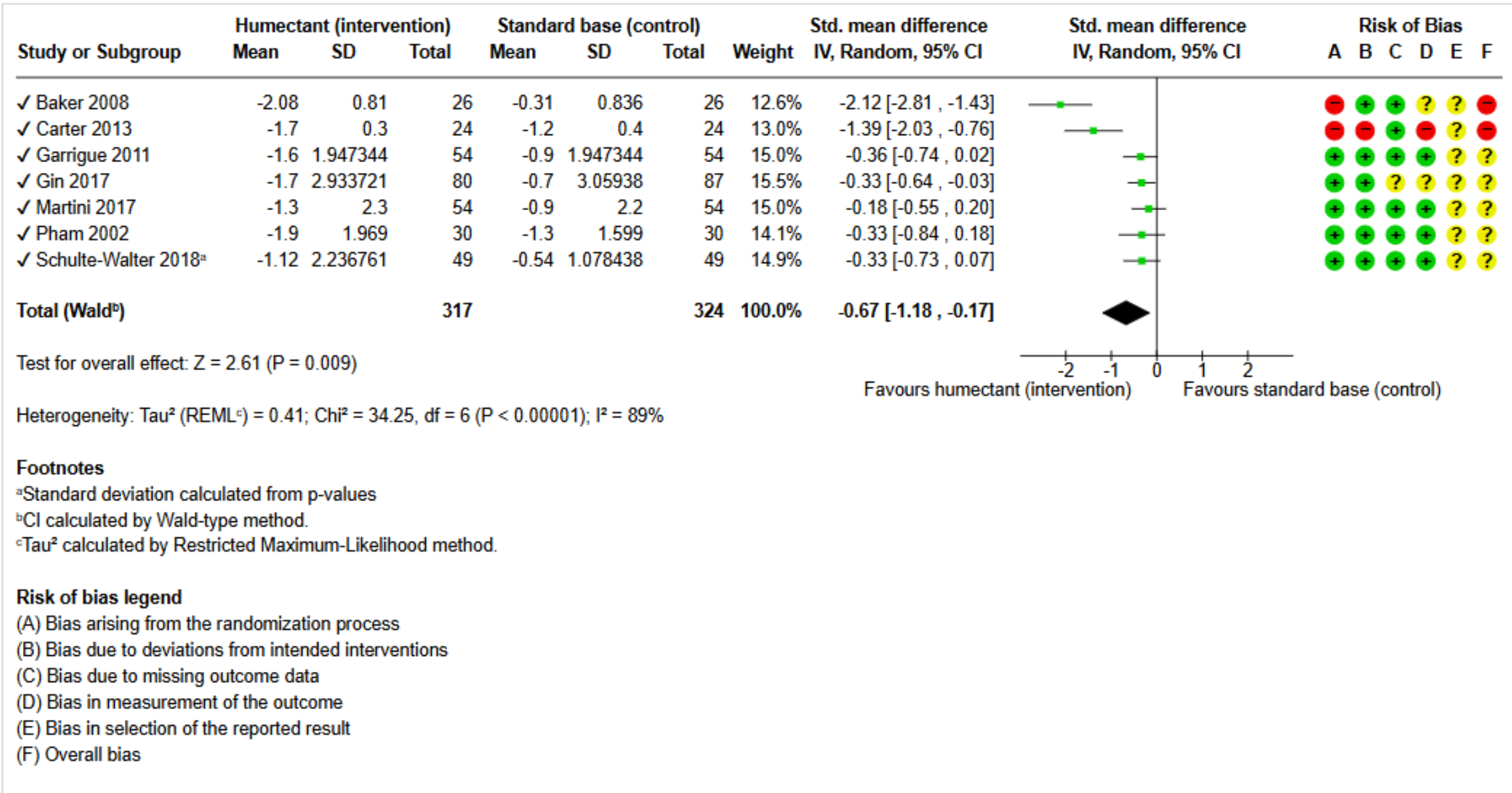

Study or Subgroup	Humectant (intervention) Mean	SD	Total	Standard base (control) Mean	SD	Total	Weight	Std. mean difference IV, Random, 95% CI	A	B	C	D	E	F
✓ Baker 2008	-2.08	0.81	26	-0.31	0.836	26	12.6%	-2.12 [-2.81 , -1.43]	−	+	+	?	?	−
✓ Carter 2013	-1.7	0.3	24	-1.2	0.4	24	13.0%	-1.39 [-2.03 , -0.76]	−	−	+	−	?	−
✓ Garrigue 2011	-1.6	1.947344	54	-0.9	1.947344	54	15.0%	-0.36 [-0.74 , 0.02]	+	+	+	+	?	?
✓ Gin 2017	-1.7	2.933721	80	-0.7	3.05938	87	15.5%	-0.33 [-0.64 , -0.03]	+	+	?	?	?	?
✓ Martini 2017	-1.3	2.3	54	-0.9	2.2	54	15.0%	-0.18 [-0.55 , 0.20]	+	+	+	+	?	?
✓ Pham 2002	-1.9	1.969	30	-1.3	1.599	30	14.1%	-0.33 [-0.84 , 0.18]	+	+	+	+	?	?
✓ Schulte-Walter 2018[a]	-1.12	2.236761	49	-0.54	1.078438	49	14.9%	-0.33 [-0.73 , 0.07]	+	+	+	+	?	?
**Total (Wald[b])**			**317**			**324**	**100.0%**	**-0.67 [-1.18 , -0.17]**						

Test for overall effect: Z = 2.61 (P = 0.009)

Heterogeneity: Tau² (REML[c]) = 0.41; Chi² = 34.25, df = 6 (P < 0.00001); I² = 89%

Footnotes

[a]Standard deviation calculated from p-values

[b]CI calculated by Wald-type method.

[c]Tau² calculated by Restricted Maximum-Likelihood method.

Risk of bias legend

(A) Bias arising from the randomization process
(B) Bias due to deviations from intended interventions
(C) Bias due to missing outcome data
(D) Bias in measurement of the outcome
(E) Bias in selection of the reported result
(F) Overall bias

Supplementary Figure 1. Change from baseline in xerosis grade after two weeks of treatment with a humectant cream versus a standard base cream.

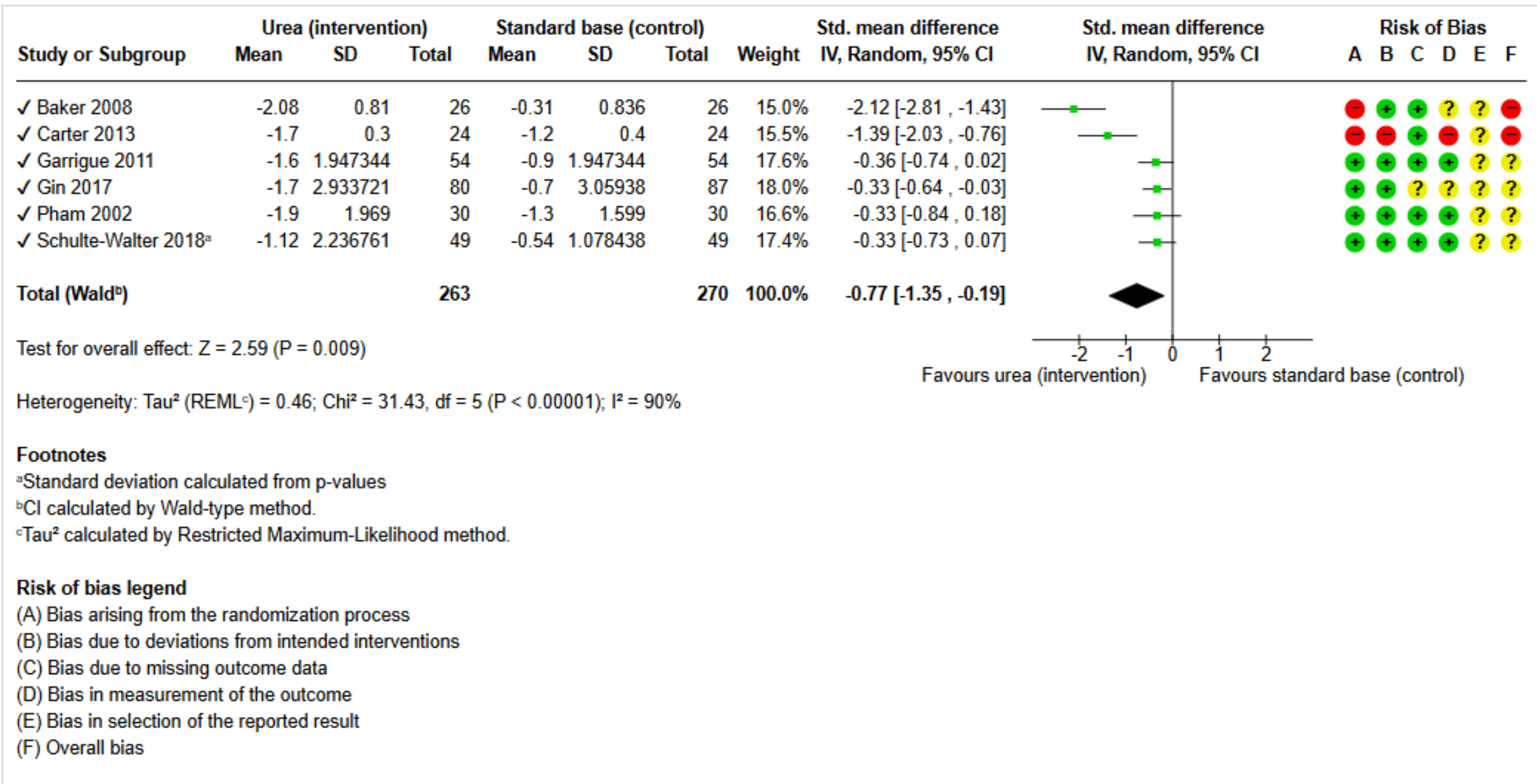

Study or Subgroup	Urea (intervention) Mean	SD	Total	Standard base (control) Mean	SD	Total	Weight	Std. mean difference IV, Random, 95% CI	A	B	C	D	E	F
✓ Baker 2008	-2.08	0.81	26	-0.31	0.836	26	15.0%	-2.12 [-2.81 , -1.43]	−	+	+	?	?	−
✓ Carter 2013	-1.7	0.3	24	-1.2	0.4	24	15.5%	-1.39 [-2.03 , -0.76]	−	−	+	−	?	−
✓ Garrigue 2011	-1.6	1.947344	54	-0.9	1.947344	54	17.6%	-0.36 [-0.74 , 0.02]	+	+	+	+	?	?
✓ Gin 2017	-1.7	2.933721	80	-0.7	3.05938	87	18.0%	-0.33 [-0.64 , -0.03]	+	+	?	?	?	?
✓ Pham 2002	-1.9	1.969	30	-1.3	1.599	30	16.6%	-0.33 [-0.84 , 0.18]	+	+	+	+	?	?
✓ Schulte-Walter 2018[a]	-1.12	2.236761	49	-0.54	1.078438	49	17.4%	-0.33 [-0.73 , 0.07]	+	+	+	+	?	?
**Total (Wald[b])**			**263**			**270**	**100.0%**	**-0.77 [-1.35 , -0.19]**						

Test for overall effect: Z = 2.59 (P = 0.009)

Heterogeneity: Tau² (REML[c]) = 0.46; Chi² = 31.43, df = 5 (P < 0.00001); I² = 90%

Footnotes

[a]Standard deviation calculated from p-values

[b]CI calculated by Wald-type method.

[c]Tau² calculated by Restricted Maximum-Likelihood method.

Risk of bias legend

(A) Bias arising from the randomization process
(B) Bias due to deviations from intended interventions
(C) Bias due to missing outcome data
(D) Bias in measurement of the outcome
(E) Bias in selection of the reported result
(F) Overall bias

Supplementary Figure 2. Change from baseline in xerosis grade after two weeks of treatment with a urea-based cream versus a standard base cream.

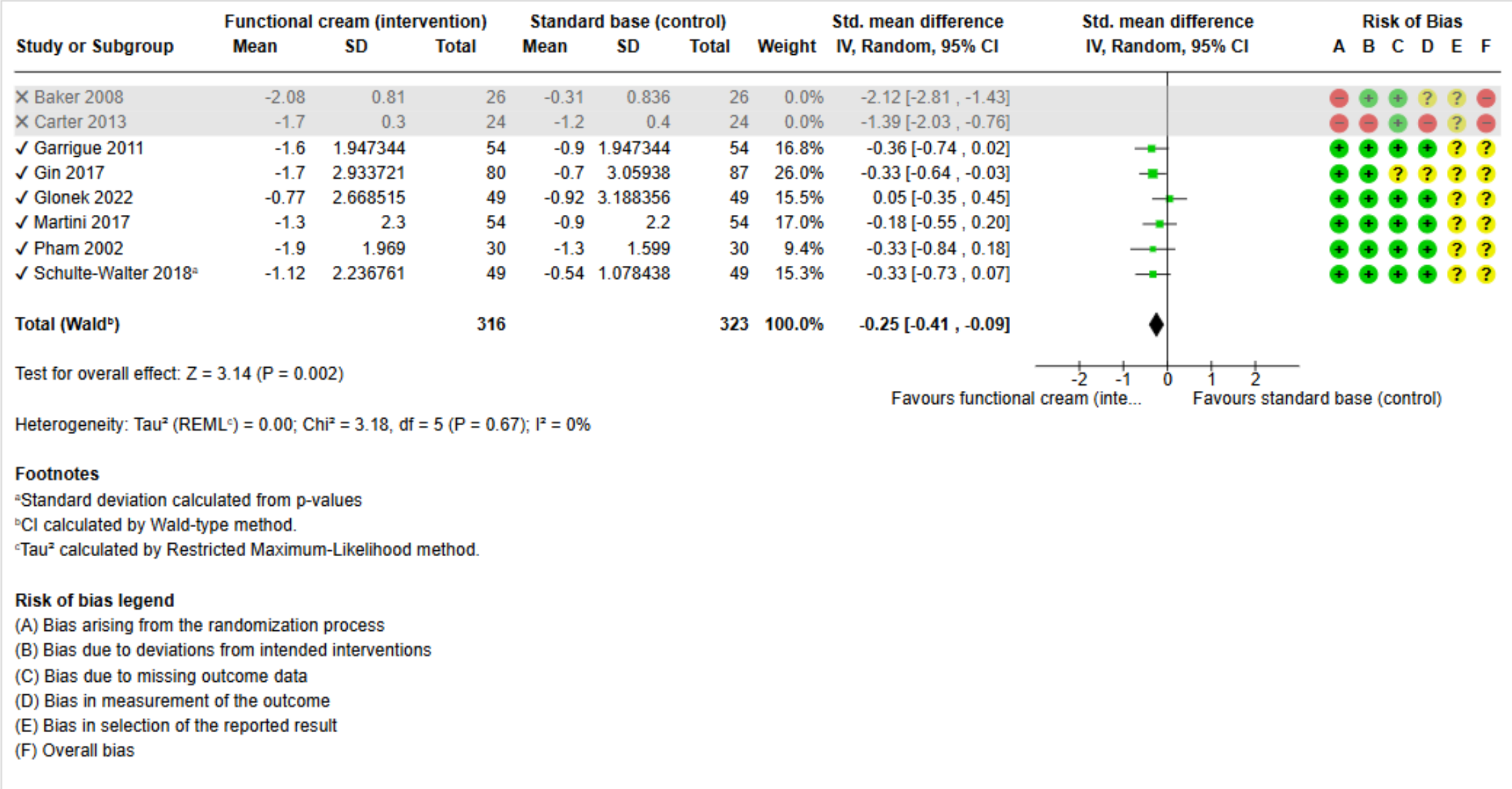

Supplementary Figure 3. Sensitivity analysis after removing studies with high risk of bias. Change from baseline in xerosis grade after two weeks of treatment with a functional cream versus a standard base cream.

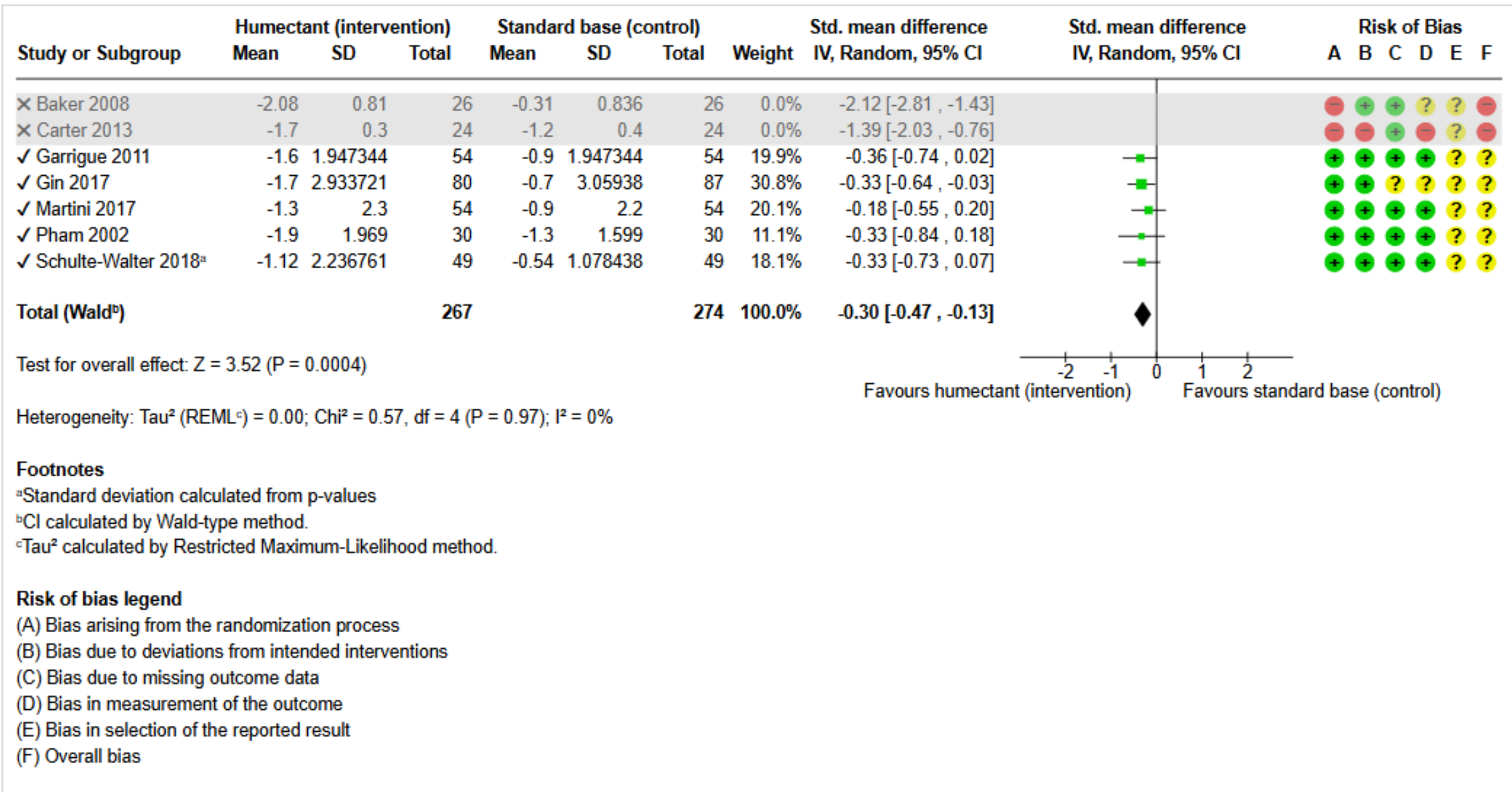

Supplementary Figure 4. Sensitivity analysis after removing studies with high risk of bias. Change from baseline in xerosis grade after two weeks of treatment with a humectant cream versus a standard base cream.

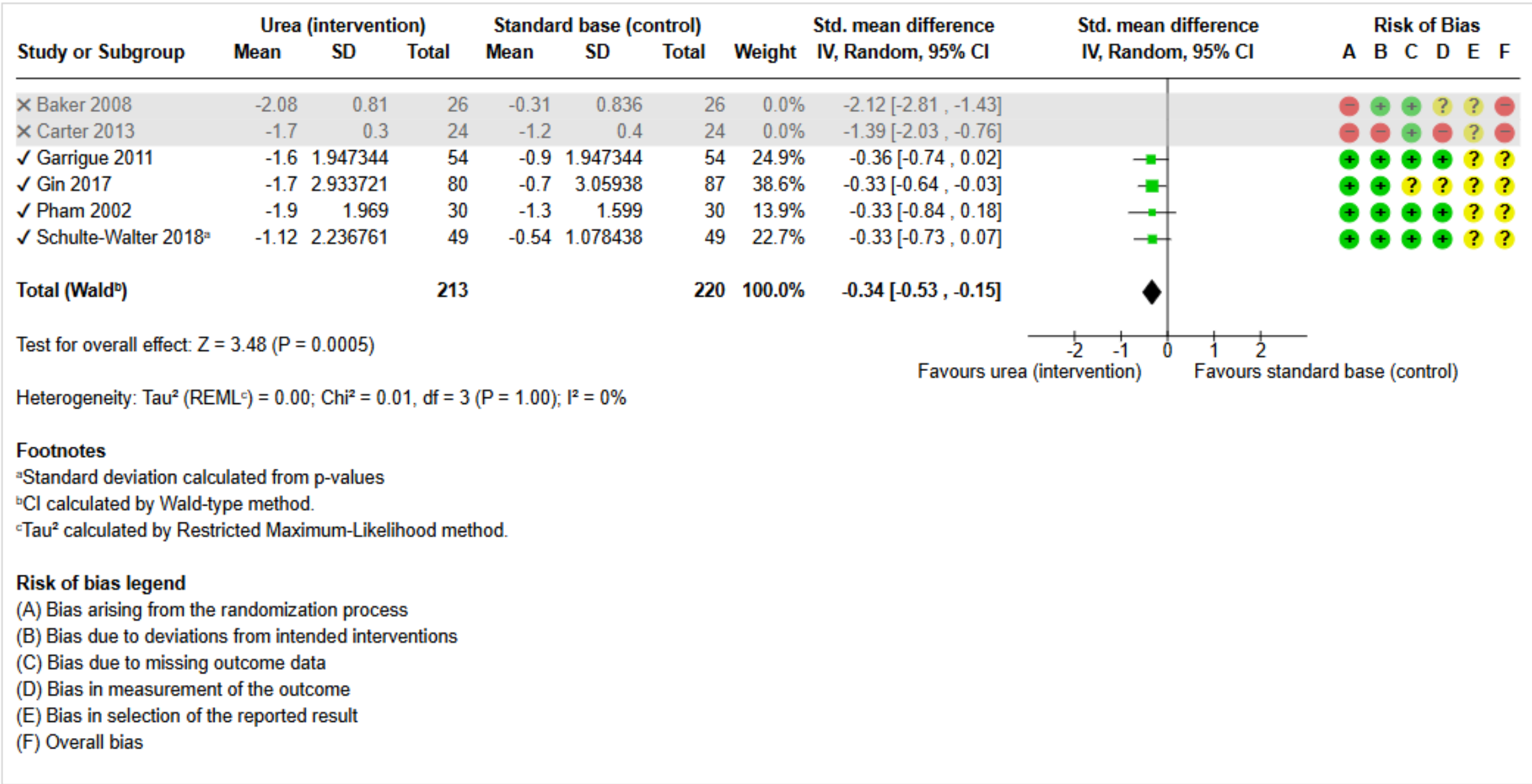

Supplementary Figure 5. Sensitivity analysis after removing studies with high risk of bias. Change from baseline in xerosis grade after two weeks of treatment with a urea-based cream versus a standard base cream.

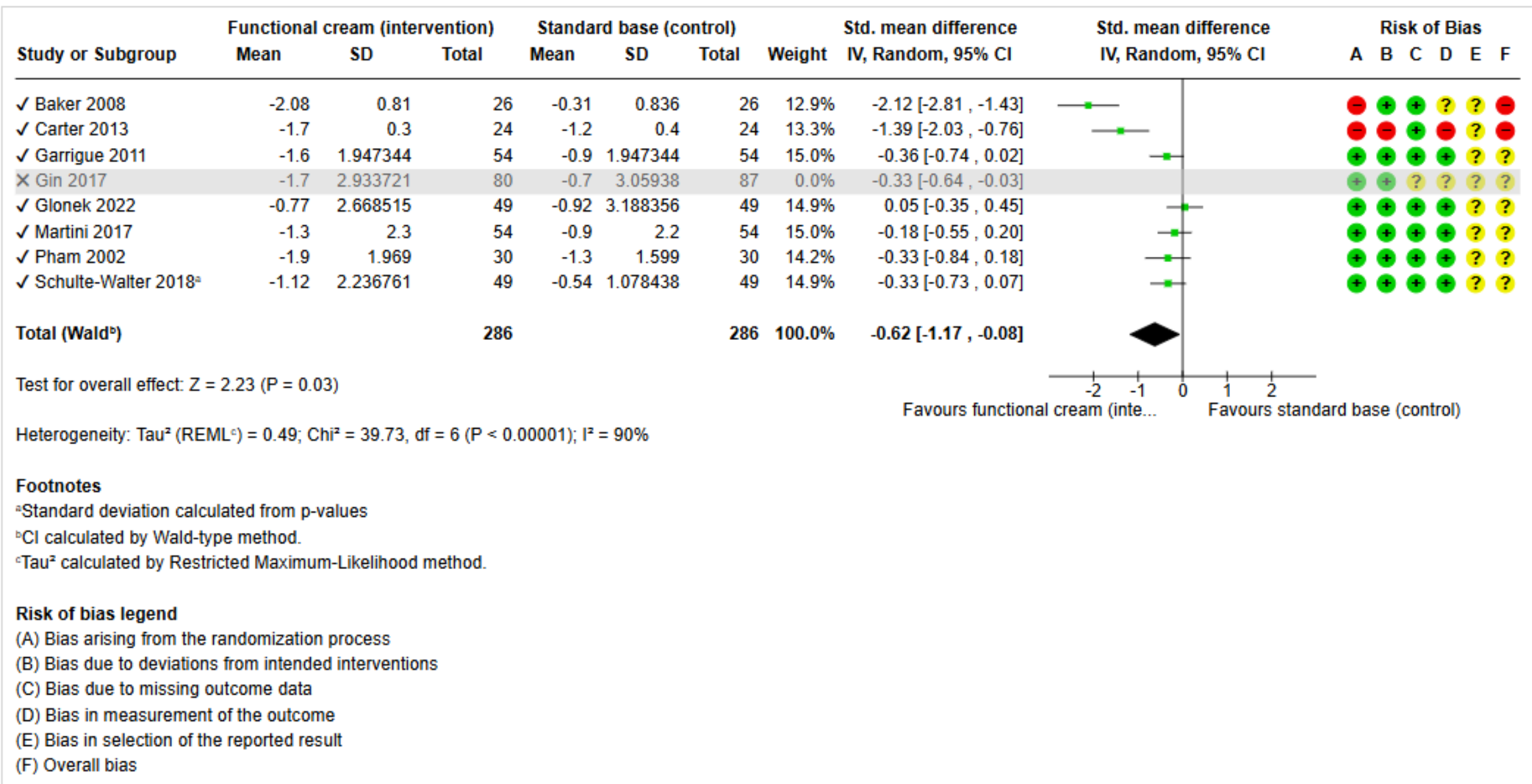

Supplementary Figure 6. Sensitivity analysis after removing studies that that randomised individuals (not within-person design). Change from baseline in xerosis grade after two weeks of treatment with a functional cream versus a standard base cream.

Study or Subgroup	Functional cream (intervention) Mean	SD	Total	Standard base (control) Mean	SD	Total	Weight	Std. mean difference IV, Random, 95% CI	Risk of Bias A	B	C	D	E	F
✓ Baker 2008	-2.08	0.81	26	-0.31	0.836	26	18.5%	-2.12 [-2.81 , -1.43]	-	+	+	?	?	-
✓ Carter 2013	-1.7	0.3	24	-1.2	0.4	24	19.0%	-1.39 [-2.03 , -0.76]	-	-	+	-	?	-
✓ Garrigue 2011	-1.6	1.947344	54	-0.9	1.947344	54	20.7%	-0.36 [-0.74 , 0.02]	+	+	+	+	?	?
✓ Gin 2017	-1.7	2.933721	80	-0.7	3.05938	87	21.1%	-0.33 [-0.64 , -0.03]	+	+	?	?	?	?
✓ Glonek 2022	-0.77	2.668515	49	-0.92	3.188356	49	20.6%	0.05 [-0.35 , 0.45]	+	+	+	+	?	?
✕ Martini 2017	-1.3	2.3	54	-0.9	2.2	54	0.0%	-0.18 [-0.55 , 0.20]	+	+	+	+	?	?
✕ Pham 2002	-1.9	1.969	30	-1.3	1.599	30	0.0%	-0.33 [-0.84 , 0.18]	+	+	+	+	?	?
✕ Schulte-Walter 2018[a]	-1.12	2.236761	49	-0.54	1.078438	49	0.0%	-0.33 [-0.73 , 0.07]	+	+	+	+	?	?
**Total (Wald[b])**			**233**			**240**	**100.0%**	**-0.79 [-1.55 , -0.03]**						

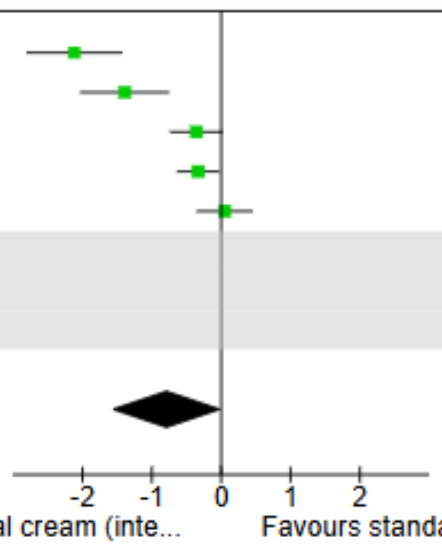

Test for overall effect: Z = 2.03 (P = 0.04)

Heterogeneity: Tau² (REML[c]) = 0.69; Chi² = 37.79, df = 4 (P < 0.00001); I² = 93%

Footnotes
[a]Standard deviation calculated from p-values
[b]CI calculated by Wald-type method.
[c]Tau² calculated by Restricted Maximum-Likelihood method.

Risk of bias legend
(A) Bias arising from the randomization process
(B) Bias due to deviations from intended interventions
(C) Bias due to missing outcome data
(D) Bias in measurement of the outcome
(E) Bias in selection of the reported result
(F) Overall bias

Supplementary Figure 7. Sensitivity analysis after removing studies for which it was unclear whether the dependency between paired observations had been fully accounted for. Change from baseline in xerosis grade after two weeks of treatment with a functional cream versus a standard base cream.

Study or Subgroup	Humectant (intervention) Mean	SD	Total	Standard base (control) Mean	SD	Total	Weight	Std. mean difference IV, Random, 95% CI	Risk of Bias A	B	C	D	E	F
✓ Garrigue 2011	-2.5	1.947344	54	-1.5	1.947344	54	19.7%	-0.51 [-0.89 , -0.13]	+	+	+	+	?	?
✓ Gin 2017	-2.4	2.719059	80	-1.7	2.798214	87	31.2%	-0.25 [-0.56 , 0.05]	+	+	?	?	?	?
✓ Martini 2017	-1.9	2.425	54	-1	2.3	54	20.0%	-0.38 [-0.76 , 0.00]	+	+	+	+	?	?
✓ Pham 2002	-2.94	1.969	30	-1.79	1.93	30	10.8%	-0.58 [-1.10 , -0.06]	+	+	+	+	?	?
✓ Schulte-Walter 2018[a]	-1.1	2.196819	49	-0.66	1.318091	49	18.3%	-0.24 [-0.64 , 0.16]	+	+	+	+	?	?
**Total (Wald[b])**			**267**			**274**	**100.0%**	**-0.36 [-0.53 , -0.19]**						

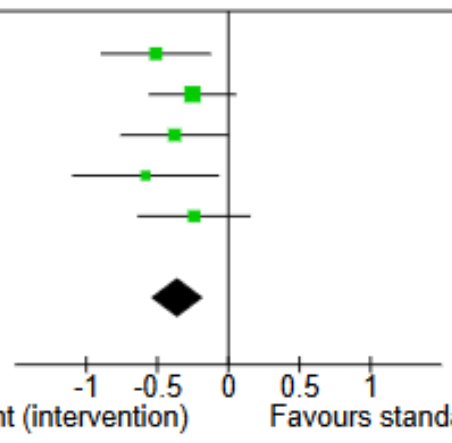

Test for overall effect: Z = 4.17 (P < 0.0001)

Heterogeneity: Tau² (REML[c]) = 0.00; Chi² = 2.13, df = 4 (P = 0.71); I² = 0%

Footnotes
[a]Standard deviation calculated from p-values
[b]CI calculated by Wald-type method.
[c]Tau² calculated by Restricted Maximum-Likelihood method.

Risk of bias legend
(A) Bias arising from the randomization process
(B) Bias due to deviations from intended interventions
(C) Bias due to missing outcome data
(D) Bias in measurement of the outcome
(E) Bias in selection of the reported result
(F) Overall bias

Supplementary Figure 8. Change from baseline in xerosis grade after four weeks of treatment with a humectant cream versus a standard base cream.

Study or Subgroup	Urea (intervention) Mean	SD	Total	Standard base (control) Mean	SD	Total	Weight	Std. mean difference IV, Random, 95% CI	Risk of Bias A	B	C	D	E	F
✓ Garrigue 2011	-2.5	1.947344	54	-1.5	1.947344	54	24.6%	-0.51 [-0.89 , -0.13]	+	+	+	+	?	?
✓ Gin 2017	-2.4	2.719059	80	-1.7	2.798214	87	39.0%	-0.25 [-0.56 , 0.05]	+	+	?	?	?	?
✓ Pham 2002	-2.94	1.969	30	-1.79	1.93	30	13.5%	-0.58 [-1.10 , -0.06]	+	+	+	+	?	?
✓ Schulte-Walter 2018[a]	-1.1	2.196819	49	-0.66	1.318091	49	22.9%	-0.24 [-0.64 , 0.16]	+	+	+	+	?	?
**Total (Wald[b])**			**213**			**220**	**100.0%**	**-0.36 [-0.55 , -0.17]**						

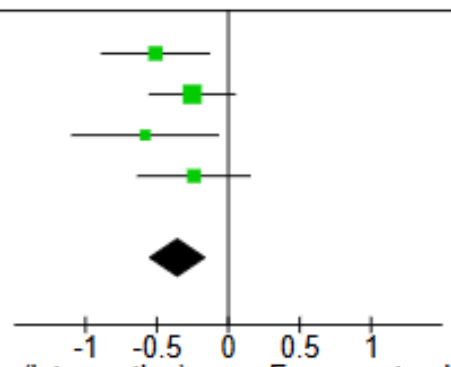

Test for overall effect: Z = 3.69 (P = 0.0002)

Heterogeneity: Tau² (REML[c]) = 0.00; Chi² = 2.12, df = 3 (P = 0.55); I² = 0%

Footnotes
[a]Standard deviation calculated from p-values
[b]CI calculated by Wald-type method.
[c]Tau² calculated by Restricted Maximum-Likelihood method.

Risk of bias legend
(A) Bias arising from the randomization process
(B) Bias due to deviations from intended interventions
(C) Bias due to missing outcome data
(D) Bias in measurement of the outcome
(E) Bias in selection of the reported result
(F) Overall bias

Supplementary Figure 9. Change from baseline in xerosis grade after four weeks of treatment with a urea-based cream versus a standard base cream.

Study or Subgroup	Experimental Mean	SD	Total	Control Mean	SD	Total	Weight	Std. mean difference IV, Random, 95% CI	Risk of Bias A	B	C	D	E	F
✓ Garrigue 2011	7.5	9.72	54	4.5	8.94	54	34.8%	0.32 [-0.06 , 0.70]	+	+	+	+	?	?
✓ Martini 2017	10.46	8.3	54	6.86	8.08	54	34.4%	0.44 [0.05 , 0.82]	+	+	+	+	?	?
✓ Schulte-Walter 2018[a]	13.54	8	49	9.19	8	49	30.8%	0.54 [0.14 , 0.94]	+	+	+	+	?	?
**Total (Wald[b])**			**157**			**157**	**100.0%**	**0.43 [0.20 , 0.65]**						

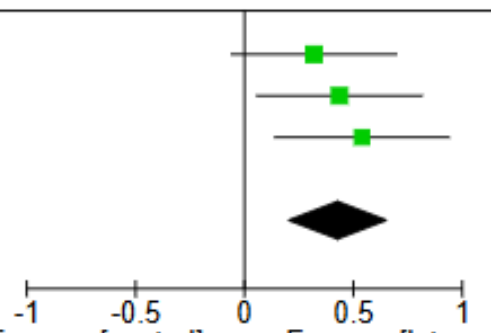

Test for overall effect: Z = 3.74 (P = 0.0002)

Heterogeneity: Tau² (REML[c]) = 0.00; Chi² = 0.61, df = 2 (P = 0.74); I² = 0%

Footnotes
[a]No SD available. Imputed from similar studies.
[b]CI calculated by Wald-type method.
[c]Tau² calculated by Restricted Maximum-Likelihood method.

Risk of bias legend
(A) Bias arising from the randomization process
(B) Bias due to deviations from intended interventions
(C) Bias due to missing outcome data
(D) Bias in measurement of the outcome
(E) Bias in selection of the reported result
(F) Overall bias

Supplementary Figure 10. Skin hydration change after four weeks of treatment measured with corneometer: Functional cream versus Standard base.

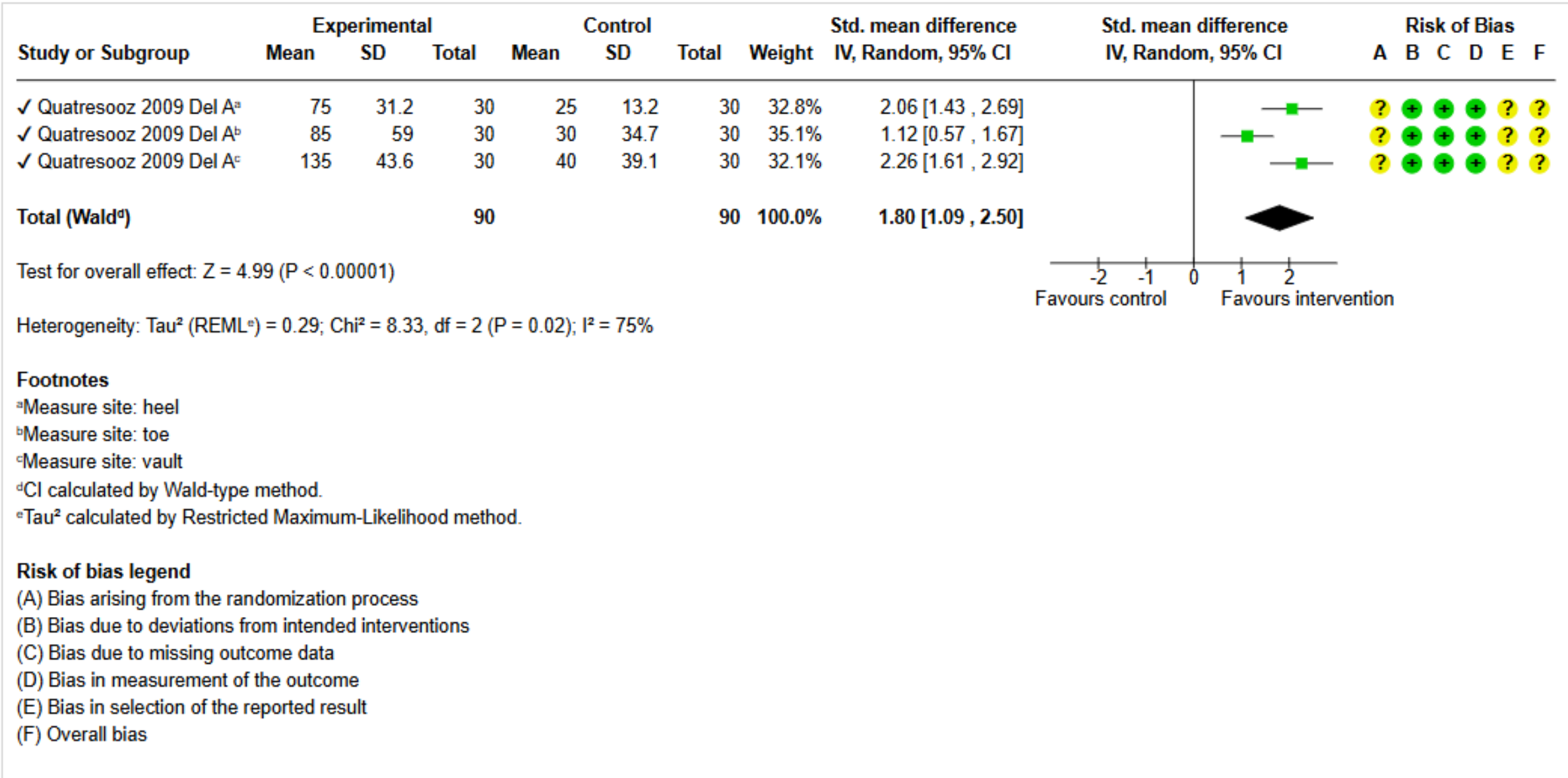

Supplementary Figure 11. Skin hydration change after two weeks of treatment measured with DPM at three different anatomical sites: Functional cream versus Standard base.

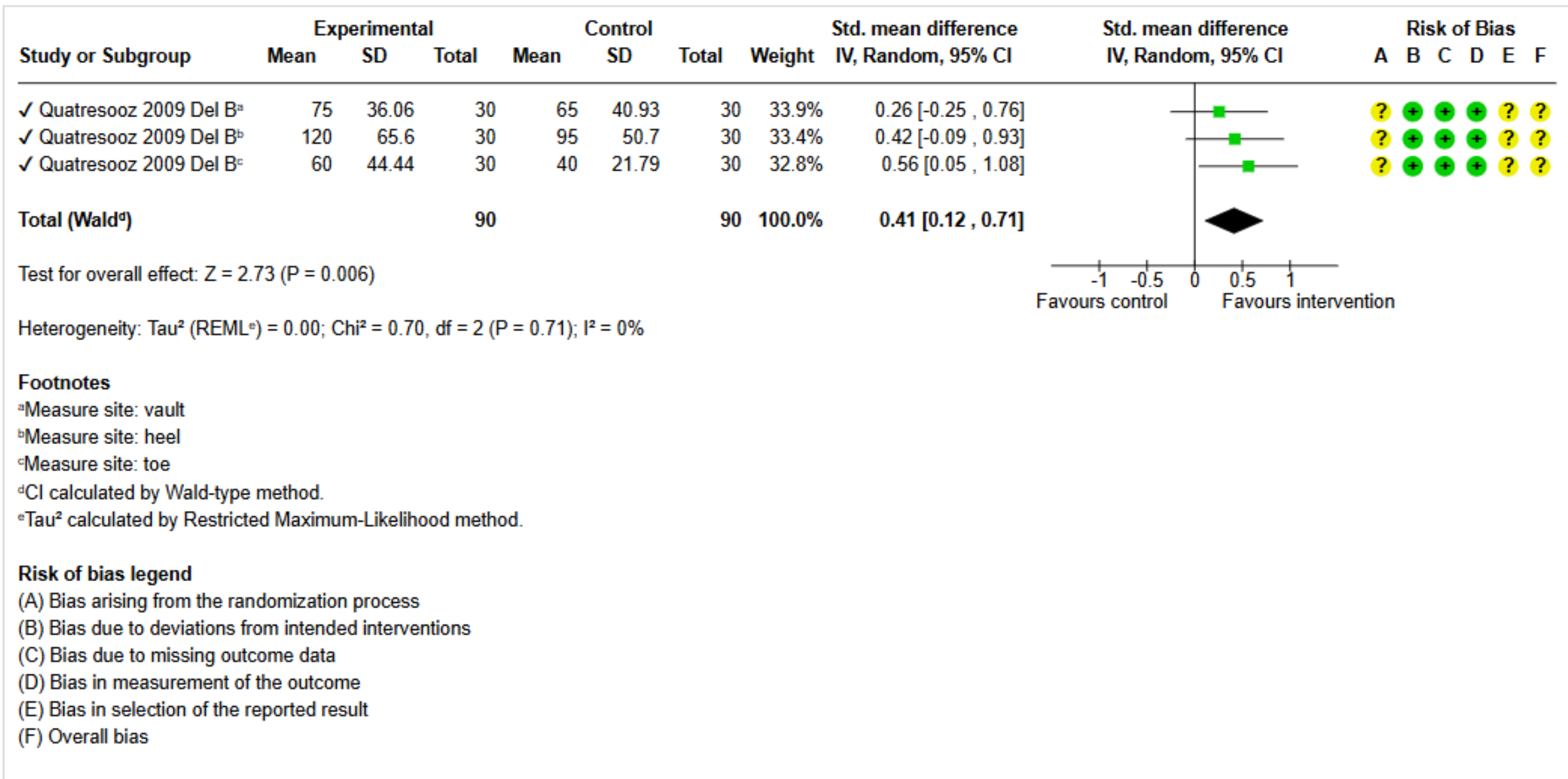

Supplementary Figure 12. Skin hydration change after two weeks of treatment measured with DPM at three different anatomical sites Humectant cream versus Humectant cream.
